# A modular design of molecular qubits to implement universal quantum gates

**DOI:** 10.1038/ncomms11377

**Published:** 2016-04-25

**Authors:** Jesús Ferrando-Soria, Eufemio Moreno Pineda, Alessandro Chiesa, Antonio Fernandez, Samantha A. Magee, Stefano Carretta, Paolo Santini, Iñigo J. Vitorica-Yrezabal, Floriana Tuna, Grigore A. Timco, Eric J.L. McInnes, Richard E.P. Winpenny

**Affiliations:** 1 School of Chemistry and Photon Science Institute, The University of Manchester, Oxford Road, Manchester M13 9PL, UK; 2 Dipartimento di Fisica e Scienze della Terra, Università di Parma, viale delle Scienze 7/a, Parma 43123, Italy

## Abstract

The physical implementation of quantum information processing relies on individual modules—qubits—and operations that modify such modules either individually or in groups—quantum gates. Two examples of gates that entangle pairs of qubits are the controlled NOT-gate (CNOT) gate, which flips the state of one qubit depending on the state of another, and the 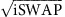 gate that brings a two-qubit product state into a superposition involving partially swapping the qubit states. Here we show that through supramolecular chemistry a single simple module, molecular {Cr_7_Ni} rings, which act as the qubits, can be assembled into structures suitable for either the CNOT or 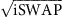 gate by choice of linker, and we characterize these structures by electron spin resonance spectroscopy. We introduce two schemes for implementing such gates with these supramolecular assemblies and perform detailed simulations, based on the measured parameters including decoherence, to demonstrate how the gates would operate.

## Abstract (web summary)

The physical implementation of quantum information processing requires individual qubits and entangling gates. Here, the authors demonstrate a modular implementation through chemistry, assembling molecular {Cr_7_Ni} rings acting as qubits, with supramolecular structures realizing gates by choice of the linker.

## Graphical abstract

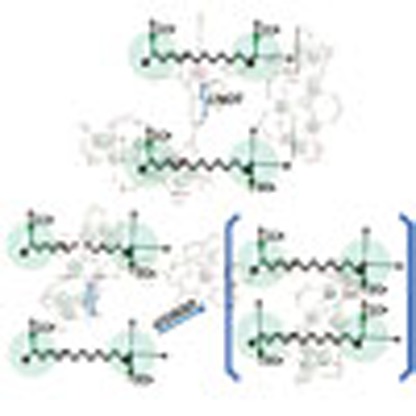

The inability of conventional computers to solve certain problems efficiently, such as the simulation of quantum systems^1^,^2^, is one of the main driving forces for the implementation of quantum computing and quantum information processing (QIP)^3^,^4^, which exploit the laws of quantum mechanics. At the theoretical level, several algorithms have been shown to outperform classical computers in certain computational tasks such as factoring large numbers into primes^5^ and searching of unsorted directories^6^. A variety of quantum systems have shown an excellent performance as the basic units for quantum information (qubits)^7^,^8^,^9^,^10^,^11^. However, they are difficult to link controllably into useful arrays producing the two-qubit entangling quantum gates (QGs) crucial for any quantum algorithm. Among the most important entangling QGs are the controlled NOT-gate (CNOT) and the 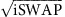 gate. The 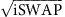 gate brings the two-qubit state 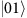 to the superposition 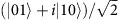. The CNOT gate flips the state of the target qubit if, and only if, the control qubit is in the 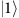 state; this implies that each qubit has to respond inequivalently to an external stimulus. All other quantum gates, however complex, can be constructed from these two-qubit gates and single-qubit gates.

Therefore the major challenge for the physical implementation of QIP is bringing together qubits in an organized, scalable and addressable way to make such QGs^3^,^4^. Here we demonstrate that supramolecular chemistry could have a major impact in addressing this challenge.

Molecular nanomagnets have been proposed as qubits^12^,^13^,^14^,^15^,^16^,^17^,^18^,^19^,^20^,^21^,^22^,^23^,^24^,^25^,^26^ and coherence times of individual molecular qubits have been studied and have improved^24^,^25^. Supramolecular chemistry^27^ allows us to bring together, with great control, complex molecules into arrays, and recently this has been exploited to tune two-qubit interactions built from molecular nanomagnets^28^. Hence supramolecular chemistry is a promising tool to build multi-qubit devices; if this could be made scalable this is a competitive route towards the realization of a quantum computer.

Demonstrations of CNOT gates based on molecular nanomagnets have been published^18^,^19^,^23^. The interaction between two dissimilar qubits produces a splitting in the low-lying two-qubit states that allows selective addressing of the transition by means of resonant EPR pulses, while keeping the other components of the wave-function frozen. In this way, the excitation of the target depends on the state of the control qubit and a CNOT gate is implemented. The drawback of this approach is the permanent direct coupling between the qubits, which makes these proposals hard to scale as with a permanent coupling the state of the qubits experiences an unwanted many-body spontaneous evolution in time (as in NMR QIP schemes^29^), whose harmful effects increase with the number of qubits. Conversely, a switchable indirect coupling between the two qubits would make the register much more easily scalable as when the switch is in the off state the unwanted spontaneous evolution is suppressed.

We have proposed using {Cr_7_Ni} heterometallic rings as qubits^14^. They are two-level systems (*S*=1/2 ground state) that have sufficiently long phase memory times to allow many gate operations before state degradation occurs^25^, and assemblies of {Cr_7_Ni} rings have been made which show a permanent coupling between the spins^26^. Here we report two-qubit assemblies which include switchable links, that allow us to propose a fully modular supramolecular design strategy^27^,^28^ towards quantum computation schemes based on either the CNOT or 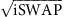 gate. The strategy uses supramolecular chemistry to tailor the individual components, spatial configuration and hence the properties of the resulting supramolecules. These supramolecules have been studied by electrochemistry, continuous-wave and pulsed EPR spectroscopy to understand their static and dynamic spin properties. These measured parameters have been used to perform detailed simulations of the performance of both the CNOT and 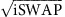 gates including the effects of decoherence. The two gates we propose are based on either global or local control of the qubit–qubit interaction. The first proposal exploits uniform magnetic pulses to manipulate two inequivalent Cr_7_Ni qubits in an asymmetric supramolecule, which would implement the CNOT gate. The second exploits the local electric control of a redox-active linker, which might be addressed by a tip to reversibly switch on and off the qubit–qubit interaction, to implement the 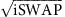 gate. The scalability of these approaches is discussed in the last part of the paper.

## Results

### Syntheses and structural characterization

**Figure 1:**
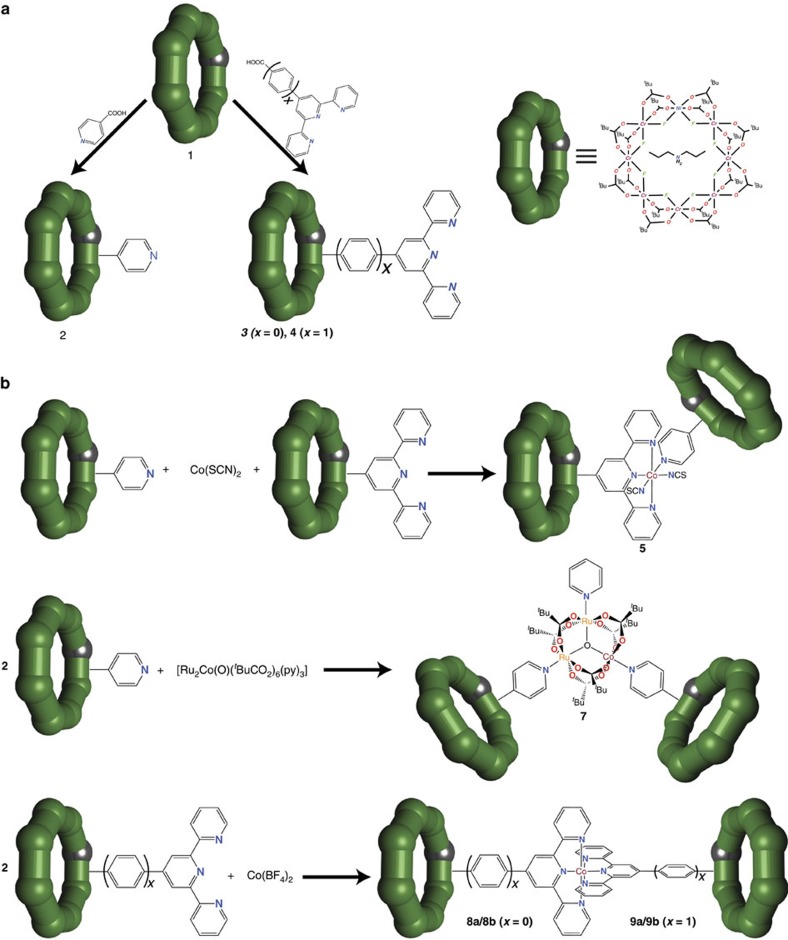
Supramolecular design strategy for the construction of two-qubits assemblies. (**a**) Synthesis of functionalized {Cr_7_Ni} rings (where *x*=0 and 1). (**b**) Linkage of {Cr_7_Ni} coordination cages to different central nodes.

We have earlier reported the selective functionalization of [^*n*^Pr_2_NH_2_][Cr_7_NiF_8_(O_2_C^*t*^Bu)_16_] **1** (ref. 30) with *iso*-nicotinate (O_2_C-py) to obtain [^*n*^Pr_2_NH_2_][Cr_7_NiF_8_(O_2_C^*t*^Bu)_15_(O_2_C-py)] **2** (ref. 26) (Supplementary Fig. 1a). This synthetic method can be extended to produce [^*n*^Pr_2_NH_2_][Cr_7_NiF_8_(O_2_C^*t*^Bu)_15_(O_2_C-terpy)] **3** and [^*n*^Pr_2_NH_2_][Cr_7_NiF_8_(O_2_C^*t*^Bu)_15_(O_2_C-Ph-terpy)] **4** (Supplementary Fig. 1b,c), from the controlled reaction of **1** with 4-carboxy-2,2′:6′,2″-terpyridine (O_2_C-terpy) and 4′-(4-carboxyphenyl)-2,2′:6′,2″-terpyridine (O_2_C-Ph-terpy), respectively (full experimental details are given in the Supplementary Methods)^31^. **2**, **3** and **4** are hereafter abbreviated as {Cr_7_Ni-O_2_C-py}, {Cr_7_Ni-O_2_C-terpy} and {Cr_7_Ni-O_2_C-Ph-terpy}, respectively. They consist of Cr^III^_7_Ni^II^ rings, containing an octagon of metal centres, with the inner rim bridged by fluoride ions and the outer rim by carboxylates: the functionalized O_2_C-py **2**, O_2_C-terpy **3** and O_2_C-Ph-terpy **4** ligands sit on a Cr…Ni edge of the octagon. This provides us with three supramolecular qubits with two different denticities, which allow us to assemble different QGs by appropriate choice of the central node (Fig. 1).

**Figure 2:**
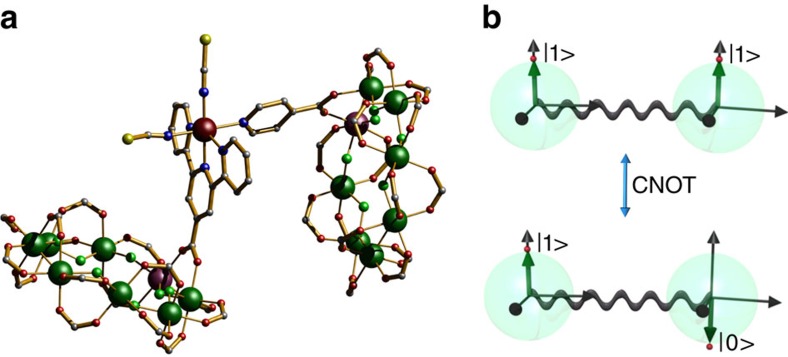
Molecular structure of the asymmetric two-qubit assembly for proposed implementation of a CNOT gate. (**a**) Molecular structure of [{Cr_7_Ni-O_2_C-py}→Co(SCN)_2_←{Cr_7_Ni-O_2_C-terpy}] **5**. ^*n*^Pr_2_NH_2_^+^ cations are not shown (H atoms and tert-butyl groups are omitted for clarity). Colour code: Co, dark red; Cr, green; Ni, purple; Ru, brown; N, cyan; O, red; S, yellow; C, grey; F, pale green. (**b**) Schematic representation of the effect of the CNOT gate on a pair of qubits, initialized in the computational basis states 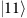 and 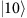, respectively. The CNOT flips the target qubit if the control is set to 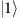.

Reaction of equimolar quantities of **2** and **3** with cobalt(II) thiocyanate in a mixture of Et_2_O/acetone leads to [{Cr_7_Ni-O_2_C-py}→Co(SCN)_2_←{Cr_7_Ni-O_2_C-terpy}] **5** in good yield, which has been characterized by X-ray single crystal diffraction (Fig. 2a; for all crystallographic information see Supplementary Data 1). Compound **5** contains two inequivalent Cr_7_Ni qubits coordinated to a central cobalt(II) ion, which has a six-coordinate CoN_6_ octahedral environment with a *cis*-arrangement of the two thiocyanate N atoms. The Co-N bond distances are typical of high spin cobalt(II) ions^32^, with the bonds to the thiocyanate ligands shorter (average 2.056(12) Å) than those to terpy or pyridine N-donors (2.137(6) to 2.224(4)  Å). Hence the d^7^ Co^II^ site (*S*_Co_=^3^/_2_) has a ^4^T_1g_ ground term (using *O*_h_ symmetry labels)^33^, which leads to a well-isolated effective *S*_eff_=^1^/_2_ ground state at low temperature (see Characterization of assemblies via EPR spectroscopy below). The *cis* coordination geometry at the Co^II^ node means the {Cr_7_Ni-O_2_C-terpy}- and {Cr_7_Ni-O_2_C-py}-based qubits are arranged in an almost orthogonal orientation. Therefore the two qubits in **5** are symmetry inequivalent, as required for implementing a CNOT gate (Fig. 2b).

**Figure 3:**
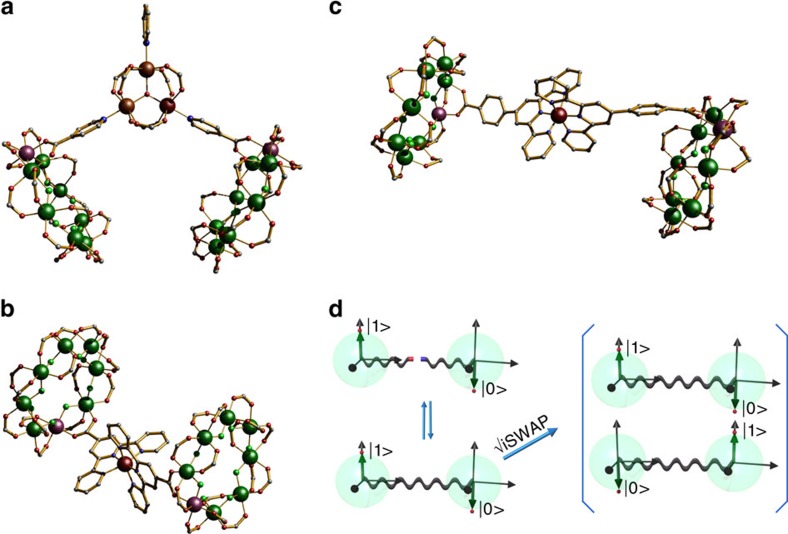
Molecular structures of the redox-active two-qubit assemblies for proposed implementation of an ✓iSWAP gate. (**a**) [{Cr_7_Ni-O_2_C-py}→[Ru^III^_2_Co^II^O(^t^BuCO_2_)_6_(py)]←{Cr_7_Ni-O_2_C-py}] **7**. (**b**) the cation of [{Cr_7_Ni-O_2_C-terpy}→Co←{Cr_7_Ni-O_2_C-terpy}][ClO_4_]_2_ **8a**. (**c**) the cation of [{Cr_7_Ni-O_2_C-Ph-terpy}→Co←{Cr_7_Ni-O_2_C-Ph-terpy}][ClO_4_]_2_ **9a**. ^*n*^Pr_2_NH_2_^+^ cations and ClO_4_^−^ anions are not shown (H atoms and tert-butyl groups are omitted for clarity). Colour codes as Fig. 2. (**d**) Schematic representation of the effect of the ✓iSWAP gate on a pair of qubits, initialized in the computational basis state 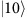. The gate brings 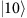 to the equal-weight superposition 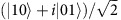. In the scheme proposed below, it operates as soon as the inter-qubit interaction is turned on (double arrow).

Reaction of two equivalents of **2** with a preformed oxo-centred pivalate-bridged triangular cluster with terminal pyridine groups [Ru^III^_2_Co^II^O(O_2_C^*t*^Bu)_6_(py)_3_] **6**, hereafter abbreviated as [Ru^III^_2_Co^II^], in acetone gives [{Cr_7_Ni-O_2_C-py}→[Ru^III^_2_Co^II^O(^*t*^BuCO_2_)_6_(py)]←{Cr_7_Ni-O_2_C-py}] **7** (Fig. 3a), where two of the terminal pyridine ligands of **6** were replaced by the *iso*-nicotinate group of **2**. Reaction of two equivalents of **3** or **4** and either cobalt(II) perchlorate or tetrafluoroborate gives [{Cr_7_Ni-O_2_C-terpy}→Co←{Cr_7_Ni-O_2_C-terpy}][X]_2_ [X=ClO_4_^−^ **8a** or BF_4_^−^ **8b**] and [{Cr_7_Ni-O_2_C-Ph-terpy}→Co←{Cr_7_Ni-O_2_C-Ph-terpy}][X]_2_ [X=ClO_4_^−^ **9a** or BF_4_^−^ **9b**] (Fig. 3b,c) (full experimental details are given in the Supplementary Methods). The architecture in **7**, **8** and **9** contains two equivalent qubits, separated by a redox-switchable centre^34^,^35^, which makes it suitable for implementation of a ✓iSWAP gate (Fig. 3d).

The crystal structure of **7** consists of two {Cr_7_Ni-O_2_C-py} rings linked through the *iso*-nicotinate groups to a [Ru^III^_2_Co^II^O(^*t*^BuCO_2_)_6_(py)] triangle (Fig. 3a)^34^,^36^. The metal ions are statistically disordered over the three sites within the triangular M_3_O unit. They have a six-coordinate, octahedral MO_5_N environment formed by four carboxylate oxygen atoms from the bridging pivalate ligands in the equatorial plane, with the central oxide and one nitrogen atom from *iso*-nicotinate or pyridine groups occupying the axial positions. The three metal ions are positioned at the corners of an isosceles triangle with two distinct intermetallic distances of 3.296 and 3.315 Å. The central oxide lies within the M_3_ plane of the metal atoms, while the six pivalate bridging ligands lie above and below the M_3_O plane and the pyridine N-donors rest perpendicular to this plane.

The crystal structure of **8a** and **9a** are made up of cationic [{Cr_7_Ni-O_2_C-terpy}Co^II^{Cr_7_Ni-O_2_C-terpy}] and [{Cr_7_Ni-O_2_C-Ph-terpy}Co^II^{Cr_7_Ni-O_2_C-Ph-terpy}] units together with counterbalancing ClO_4_^−^ anions (Fig. 3b,c). Both compounds have a central cobalt(II) ion with a six-coordinate CoN_6_ octahedral environment, with the six Co-N bonds in the range expected for a low spin cobalt(II) ion^37^. The average value of the two N-donors from the central pyridines of the terpy are shorter (1.923(6) **8a** and 1.920(8) **9a** Å) than the other Co-N contacts (2.118(7) **8a** and 1.986(9) **9a** Å). This feature leads to an axially compressed octahedral environment for the low-spin d^7^ Co^II^ ions giving a *S*_Co_=^1^/_2_ ground state. Compounds **8a** and **9a** differ in the shortest Co…M(ring) contact (8.675(2) **8a** and 10.979(5) **9a** Å) and the staggered **8a** or eclipsed **9a** arrangement of rings in the assemblies.

The two Cr_7_Ni qubits in each of **7**–**9** are linked by a redox-switchable central node^34^,^38^. Cyclic voltammetry on **7**–**9**, and on the central nodes in isolation, show a one-electron reversible oxidation in each case (measured in CH_2_Cl_2_, 0.1 M *n*Bu_4_NPF_6_). For **6** and **7** the half-wave potential (*E*_1/2_)=−0.31 (**6**), −0.35 (**7**) V versus Fc^+^/Fc (Fc=ferrocene)^39^, and this oxidation is assigned to the [Ru^III^_2_Co^II^] to [Ru^III,IV^_2_Co^II^] couple^34^. For **8b**, **9b** and reference complexes [Co(HO_2_C-terpy)_2_][BF_4_]_2_ **10** and [Co(HO_2_C-Ph-terpy)_2_][BF_4_]_2_ **11**, *E*_1/2_=−0.30 (**8b**), −0.27 (**9b**), −0.27 (**10**) and −0.24 (**11**) V, which are assigned to the Co^II/III^ couple. The anodic to cathodic peak separation values are similar to those of ferrocene under the same conditions (see Electrochemistry section in Supplementary Methods, Supplementary Fig. 4 and Supplementary Table 3).

### Characterization of assemblies via EPR spectroscopy

To study the interactions between the molecular components in the supramolecular structures **5**, **7**, **8b** and **9b** we performed magnetometry and multi-frequency continuous wave EPR studies. The measured magnetometry data are essentially the sum of the Cr_7_Ni qubits and the linking nodes (Supplementary Figs 5–9), and therefore are uninformative other than confirming that the interactions are very weak. EPR spectroscopy is much more sensitive to such weak interactions.

**Figure 4:**
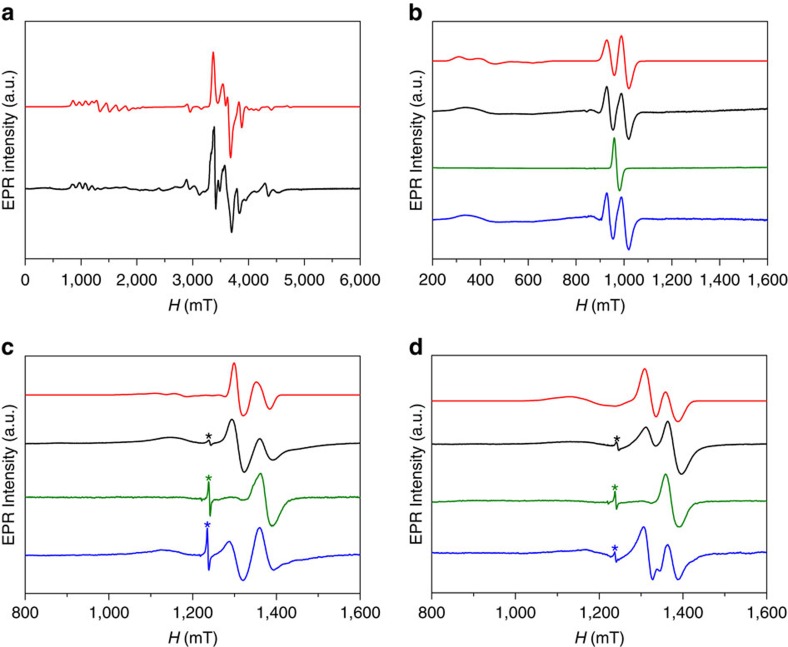
EPR spectroscopy of QG assemblies. (**a**) Experimental powder W-band (94 GHz) EPR spectrum of **5** at 5K (black) and simulation (red) using Hamiltonian (1) and parameters in Table 1. (**b**) Experimental K-band (24 GHz) EPR spectra of **7** frozen solution (black), and simulation (red) using Hamiltonian (2) and parameters given in the text: experimental spectra after oxidation of **7** to **7b**^**ox**^ with [FeCp_2_](PF_6_) (ca. 3 mM, 1:1) (green), and after reducing **7b**^**ox**^ with cobaltocene (3 mM, 1:1) (blue). (**c**,**d**) Experimental Q-band (34 GHz) EPR spectra of **8b** and **9b**, respectively, in frozen solution (black), and simulations (red) using Hamiltonian (2) and parameters given in the text; experimental spectra after oxidation to **8b**^**ox**^ and **9b**^**ox**^ with AgBF_4_ (ca. 3 mM, 1:1) (green), and after reducing using with cobaltocene (3 mM, 1:1) (blue). The sharp peak marked * is a trace radical impurity.

The EPR spectra of a powder of **5** at 5K (Fig. 4a and Supplementary Fig. 10) have a complex multiplet structure because the *S*_eff_=1/2 of the Co(II) node has a very anisotropic effective *g*-tensor and also gives rise to very anisotropic exchange tensors (*J*) with the {Cr_7_Ni} rings. This structure is best resolved at W-band (94 GHz; Fig. 4a). The spectra can be simulated^40^ with the effective three-spin Hamiltonian (1), with two different anisotropic exchange interactions (*J*_12_ and *J*_23_) between the *S*_eff_=½ of the Co(II) ion (*S*_2_) and the two *S*=½ of {Cr_7_Ni-O_2_C-terpy} (*S*_1_) and {Cr_7_Ni-O_2_C-py} (*S*_3_) rings, respectively (Fig. 4a).


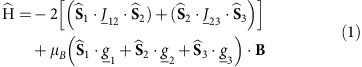


The multiplets centred at ca. 1, 1.5 and 3.5 T (Fig. 4a) mark the effective *g* values of the Co(II) ion as *g*=6.50, 4.25 and ca. 2, while the Cr_7_Ni rings have well defined *g*=1.78, 1.78 and 1.74 (the unique value being for the orientation perpendicular to the ring)^30^. The large difference (Δ*g*) between the *g*=6.5 and 4.3 orientations of the Co(II) ion and those of the rings, and the weak exchange interactions 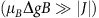 is useful as it allows us to treat the problem as an *ABX* spin system. The multiplet structure of the *g*=6.5 and 4.3 features is then due to weak exchange with two different *S*=1/2, giving doublets-of-doublets from which we can read the *J*_*y*_ and *J*_*z*_ components of *J*_12_ and *J*_23_. The high field region is more complicated, due to the much smaller difference between the third Co(II) *g* value and those of the {Cr_7_Ni} rings. The remaining parameters (the final components of *J*_12_, *J*_23_ and *g*_2_) were obtained by simulation (Fig. 4a). The parameters in Table 1 are labelled to a common reference frame, that is, taking into account the orthogonal orientation of the two {Cr_7_Ni} rings.

**7**, **8b** and **9b**, contain redox-active linking nodes and their EPR spectra change with the oxidation state. Hence, EPR was performed on both the reduced and oxidized forms at 5K as frozen solutions, allowing cycling of the redox state (Fig. 4b–d). Spectra were measured on the as prepared samples, then solutions were warmed to room temperature and the oxidized forms **7**^**ox**^, **8b**^**ox**^ and **9b**^**ox**^ generated *in situ* by addition of [FeCp_2_](PF_6_) or AgBF_4_ followed by freezing and measurement. To complete the cycle, the solutions were thawed again and **7**, **8b** and **9b** regenerated by reduction with cobaltocene. We have also measured spectra of the isolated nodes in the paramagnetic Co(II) form, viz. complex **6**, and [Co(HO_2_C-terpy)_2_](BF_4_)_2_ **10** and [Co(HO_2_C-Ph-terpy)_2_](BF_4_)_2_ **11** (Supplementary Figs 2 and 3). Like **5**, **6** also contains a ^4^T high-spin Co(II) ion with an *S*_eff_=1/2 ground state, giving *g*=5.61, 4.05, 2.77 (Supplementary Fig. 11), while **10** and **11** have low-spin Co(II) hence *S*=1/2, giving *g*=2.047, 2.076, 2.195 (Supplementary Fig. 12) and *g*=2.022, 2.111 and 2.215 (Supplementary Fig. 13), respectively.

For the supramolecular structures **7**, **8b** and **9b**, the exchange coupling is nicely resolved in K- and Q-band (24 and 34 GHz) EPR spectra (Fig. 4b–d). In each case there is weak coupling with respect to the difference in Zeeman energies 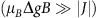
41. This, and the equivalence of the two Cr_7_Ni rings, gives *AB*_2_ spin systems. The {Cr_7_Ni} ring resonances (the ‘*B*' spins) are split into doublets (Fig. 4b–d), giving a direct measure of *J* (the exchange splittings of the central node resonances are not resolved). The spectra can be simulated with the simple Hamiltonian (2) using an isotropic exchange interaction between the central nodes (*S*_2_) and the two {Cr_7_Ni} rings (*S*_1_ and *S*_3_), even for **7,** which has an effective spin 1/2 centre at the node.


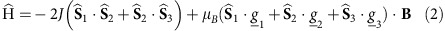


Simulations with the isotropic exchange interaction *J* as the only variable gives *J*=−0.026, −0.026 and −0.024 cm^−1^ for **7**, **8b** and **9b**, respectively; the *g* values were fixed to those measured for individual components. The EPR spectra of **7**^**ox**^, **8b**^**ox**^ and **9b**^**ox**^ are very simple, resembling isolated {Cr_7_Ni} rings, because the oxidized forms of the central nodes are diamagnetic. Reduction of **7**^**ox**^, **8b**^**ox**^ and **9b**^**ox**^ back to **7**, **8b** and **9b** regenerates the original spectrum.

Implementing interesting quantum algorithms requires qubits with long phase memory times, such that they can be manipulated many times without errors. Individual {Cr_7_Ni} heterometallic rings and simpler paramagnetic centres have shown long enough phase memory times (*T*_M_)^21^,^22^,^24^,^25^ to perform coherent electron spin manipulations before state degradation occurs. To check that this key property is preserved when they are incorporated in the supramolecular assemblies, we have performed pulsed EPR measurements at resonances corresponding to both the central nodes and the {Cr_7_Ni} rings (Supplementary Figs 14–26). For the latter we find similar values for all compounds, and in both oxidation states where relevant (*T*_M_=683, 749, 767, 790, 984, 750 and 1,031 ns for **5**, **7**, **7**^**ox**^, **8b**, **8b**^**ox**^, **9b** and **9b**^**ox**^, respectively, measured at Q-band and 3K; Supplementary Table 4), demonstrating that the phase memory times are not strongly influenced by structural or magnetic differences in the linkage of the rings. In addition, values of *T*_*M*_ measured at resonances fields corresponding to the central node (in the paramagnetic state) are also similar for all compounds (500–700 ns at Q-band and 3K; Supplementary Tables 5–12). Hence, the phase memory time of the central node does not represent a limitation (see also the Discussion below) for the applicability of our schemes for implementing a universal set of quantum gates. These results are used in the simulations below, and are extremely promising, as these *T*_M_ times are sufficiently long for spin manipulation even when they are integrated in a supramolecular assembly.

### CNOT gate with uniform magnetic pulses

**Figure 5:**
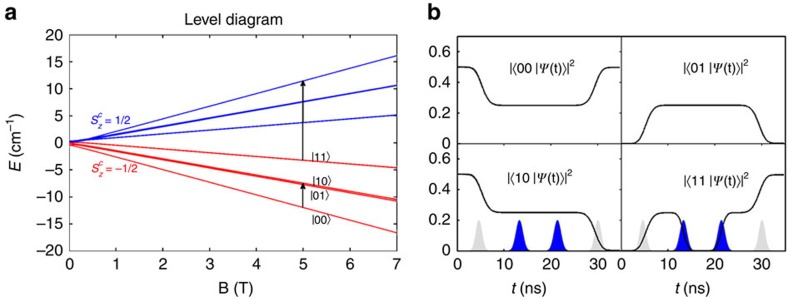
A CNOT gate based on the structure of 5. (**a**) Field-dependence of the energy levels of **5** resulting from the Hamiltonian (1). The low-energy group of levels (red), where Co is frozen into its *S*_*z*_=−1/2 state, defines the computational basis. The high-energy group of levels, where the Co spin is inverted, is exploited to perform two-qubit gates. (**b**) Simulation of the pulse sequence implementing CNOT as *R*_*y*_(*π*/2)*C*_*Z*_ *R*_*y*_(*−π*/2), where *R*_*y*_(*α*) is a rotation of the target qubit by an angle *α* around the *y* axis and *C*_Z_ is a controlled-Z gate. We illustrate the gate by starting at time *t*=*0* with a superposition state 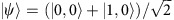, which transforms under a CNOT gate (with the left qubit acting as control) into 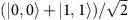. The latter state is actually obtained by the pulse sequence implementing *R*_*y*_(*π*/2)*C*_Z_*R*_*y*_(−*π*/2), with a fidelity of 99.7%. The envelope of the pulses implementing the two *R*_*y*_ rotations and the *C*_Z_ are outlined at the bottom. Note that performing the *C*_Z_ gate (two central pulses) requires temporarily leaving the computational subspace. The intensity of the oscillating field at the pulse maximum is 50 G, and we assume a static field of 5 T directed along *z*.

In the following, we introduce two quantum computation schemes, based on either local or non-local control of the inter-qubit interaction employing the structures described and the parameters obtained by EPR spectroscopy. First, we show that compound **5** is suitable for a CNOT gate, using uniform magnetic pulses as the only manipulation tool^42^. As we are treating three interacting inequivalent doublets this produces 2^3^ energy levels. We define the computational basis within the low-energy subspace where Co^II^ is frozen into its *S*_*z*_=−1/2 state, which corresponds to the four lowest levels shown in red in Fig. 5a. The four levels correspond to arrangements of the spins of the two qubits, *S*_1_ and *S*_3_, having the relative orientations |↓↓>, |↓↑>, |↑↓> and |↑↑>, respectively, which we label as |00>, |01>, |10> and |11> in Fig. 5a.

In a field of a few Teslas the eigenstates are factorized, that is, the eigenfunctions of the rings and the Co^II^ are not entangled. Hence it is possible to implement high-fidelity single-qubit rotations by EPR pulses resonant with low-energy gaps (see, for example, the shorter arrow in Fig. 5a). The combination of the inequivalent and anisotropic ring-Co exchange interaction and of the perpendicular arrangement of the two rings makes the two qubits significantly inequivalent. In particular, there is a sizeable gap (about 33 μeV in a field of 5 T) between the |00>→|01> and |00>→|10> transitions, which enables independent single-qubit rotations. We note that a distribution of the spin Hamiltonian parameters arising from a reasonable *g* or *J* strain (∼1%, see ref. 43) would yield a broadening of the energy levels with s.d. ∼5 μeV, thus keeping the two transitions distinguishable in realistic conditions, possibly with the help of narrow and/or composite pulses^44^,^45^.

A controlled phase-shift (*C*_*φ*_) gate is obtained by a pulse resonant with the transition corresponding to the longer arrow in Fig. 5a where the cobalt(II) ion is temporarily in its *S*_*z*_=*+*1/2 state (blue energy levels), followed by a repetition of the same pulse that would bring the state back with an additional phase *φ.* The value of *φ* is controlled by the phase difference between the first and the second pulse. For a pulse with field along the *y* direction, the implementation of this gate is fast, owing to the large value of *g*_*y*_ for Co^II^ ion. Moreover, the relatively large exchange interaction in equation (1) allows us to employ large oscillating fields (50 G), since the desired transition is spectroscopically well resolved from all the others. Consequently, the *C*_*φ*_ gate can be performed in only about 12 ns, with fidelities close to 99.99 % (see Computational Details section in Supplementary Methods).

The CNOT gate can then be obtained by the sequence of gates *R*_*y*_(*π*/2) *C*_*Z*_ *R*_*y*_(*−π*/2), where *R*_*y*_(*α*) is a rotation of the target Cr_7_Ni qubit by an angle *α* around the *y* axis (a single-qubit operation) and where *C*_*Z*_ is the phase-shift gate described above with *φ*=*π*. We have numerically solved the time-dependent Schrödinger equation for the Hamiltonian (1) in presence of this pulse sequence. Results are reported in Fig. 5b and show that this two-qubit gate can be obtained with very high fidelity in only 30 ns. As noted above, the two qubits are significantly inequivalent, even if the *g* tensor of the rings is nearly isotropic. This makes this complex well suited for the quantum simulation of antisymmetric Hamiltonians (Supplementary Figs 28 and 29 for an illustrative example).

### 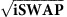 gate with local electric control

The redox properties of the central node in **7**, **8** and **9** can be exploited to perform the universal 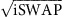 gate on the {Cr_7_Ni} qubits, whose effect on the computational basis is given by:


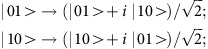



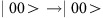
 and 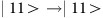.

When the central node is in the diamagnetic state ([Ru^III,IV^_2_Co^II^] **7** or Co^III^ **8**–**9**), the two qubits are decoupled and only single-qubit operations can be performed. Conversely, when the paramagnetic oxidation state, [Ru^III^_2_Co^II^] **7** or Co^II^ **8**–**9**, is present the system behaves as a trimer described by the Hamiltonian (2). Previously, Lehmann *et al*.^16^ have proposed that two spins *S*=1/2 connected by a redox-active unit can be exploited for the implementation of the 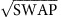 gate by switching the redox unit with a scanning tunnelling microscope (STM) tip at an appropriate potential. In this way one electron can be added or removed from the redox unit very quickly. It was demonstrated that particular sets of parameters of the trimer Hamiltonian lead to a pure 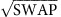 evolution of the two qubits after specific time intervals. However, high fidelity for the gate is guaranteed only for fixed ratios between the qubit–qubit exchange (*J*_qq_) and the qubit-redox unit exchange (*J*_qr_). For the measured parameters of **7**, **8** and **9** (*J*_qr_≡*J* and *J*_qq_=0 in equation (2)) the fidelity of the 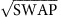 gate would be low.

Here we propose a more flexible scheme for the 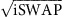 gate based on our measured parameters, which works if the two qubits have the same Zeeman energy but one different to that of the switch in the ‘on' state. This is the case here, where the *g* values of the qubits and switches are very different, *g*_1*z*_=*g*_3*z*_=1.74 for {Cr_7_Ni} rings and *g*_2*z*_=2.77 (**7**), 2.195 (**8**) and 2.215 (**9**) for the central node. This difference in the *g* values means that in magnetic fields of a few T, the ring-central node exchange *J* is small compared with the difference between the Zeeman energies of the ring and the central node. Hence, the spin state of the central node is nearly frozen in the *S*_*z*_=−1/2 state and has only tiny virtual fluctuations that lead to an effective interaction between the two {Cr_7_Ni} qubits given by


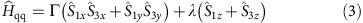


where the field is along *z*, *S*_1_ and *S*_3_ refer to the spins of the first and second Cr_7_Ni qubits and


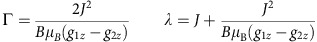


The feasibility of the scheme relies only on the equivalence of the qubits and on the hierarchy of the interactions 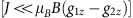 (see Supplementary Methods for more details).

Therefore when the central node is paramagnetic, the state of the two qubits evolves according to (3), with negligible entanglement with the central node. For specific times, this evolution coincides with the 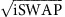 gate apart from single-qubit rotations along *z* due to the second term in (3).

**Figure 6:**
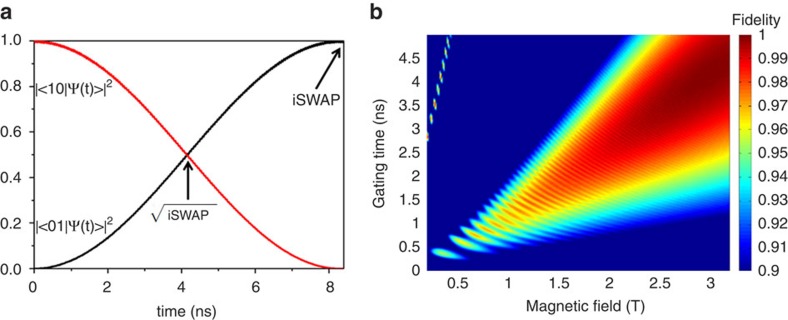
Simulation of the 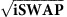 gate. (**a**) For **9**, taking |10>≡|1/2,−1/2>⊗|−1/2>_Co_ as the initial state and an applied field of 3 T, we calculate the time-dependence of the oscillation of the trimer wavefunction between |10> and |01>≡|−1/2,1/2> ⊗|−1/2>_Co_. Other components are negligible. (**b**) Calculated average fidelity 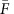 for **9** as a function of the magnetic field *B* and of the gating time, that is, the time the Co switch is in the on state. The fidelity is defined by 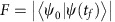, where for a given starting logical state, 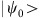 is the final state after an ideal gate, whereas 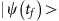 is the actual final state. The average has been made over four random starting states. For each value of the field, the optimal gating time *t*_*f*_ is the one maximizing 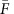 ca. 4 ns for 3 T as shown in **a**. The oscillations corresponding to the fringes in the picture are associated with fluctuations of the Co spin state. As long as the frequency of these fluctuations is much larger than 1/*t*_*f*_ these fluctuations are negligible, that is, the perturbative description of equation (3) is valid.

This perturbative picture is confirmed by the results of detailed calculations for compound **9** using the full Hamiltonian (2) (Fig. 6). Starting from the |10> logical state, we report in Fig. 6a the time evolution of the trimer wavefunction that would implement the 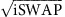 gate. In a magnetic field of 3 T, after 4 ns the wavefunction has equal-weight contributions from |10> and |01>, which is the 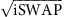 gate, while after 8 ns the states of the two spins are exchanged, that is, we have the |01> state. An extremely good fidelity *F* (larger than 0.99) for compound **9** is obtained for fields of the order of about 2.5 T or larger, after a suitable gating time *t*_*f*_ of the order of a few nanoseconds (Fig. 6b). For such fields the perturbative picture of equation (3) holds very well, and *t*_*f*_ is proportional to *B* (Fig. 6b), which is consistent with the form of the effective qubit–qubit coupling Γ∝1/*B*.

Analogous or better fidelities can be obtained with the parameters derived for compounds **7** and **8**. Indeed, larger values of |*g*_1*z*_−*g*_2*z*_| (as in **7**) increase the validity of the perturbative picture (3), even for smaller values of *B*. Hence, we can exploit the modular strategy and optimize the performance of the gates by targeted chemical manipulations.

## Discussion

Our initial simulations above did not include decoherence. To gain a deeper insight into the performance of the proposed quantum gates, we have performed further simulations that include the effect of both relaxation and pure dephasing, allowing for the finite and measured values of *T*_1_ and *T*_M_, respectively. For the values of *T*_1_ measured by pulsed EPR (>10 μs in all compounds, see the Supplementary Tables 4–12), the effect of relaxation is found to be completely negligible.

Pure dephasing is accounted for by numerically solving the Lindblad master equation for the system density matrix *ρ* (refs 46, 47):


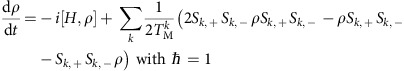


Here the commutator describes the coherent evolution induced by the full system Hamiltonian *H*, while the second term describes pure dephasing mechanisms. The subscript *k*=1, 2 and 3 labels the spins of the qubits and of the switch, while 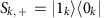 and 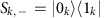 are spin 1/2 raising and lowering operators.

Using the values of *T*_M_ of the Cr_7_Ni qubits measured by pulsed-EPR spectroscopy (between 700 and 1,000 ns, see above) we still find very high fidelities: 99.3% for the implementation of the CNOT gate on compound **5** and 99.6% for the 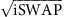 gate on **9**. As expected, the fidelities are only marginally affected by decoherence because gating times are much shorter than *T*_M_.

**Figure 7:**
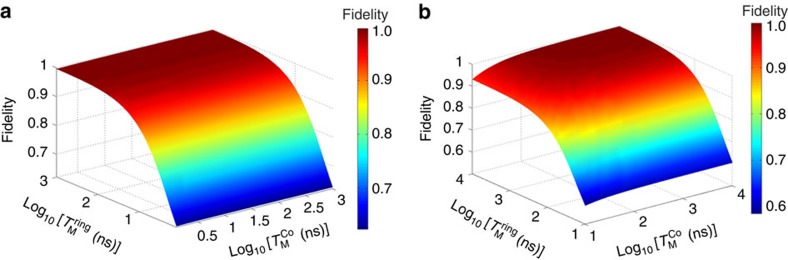
Effect of decoherence on quantum gates. (**a**) Fidelity of the 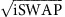 as a function of the dephasing times of the rings and of the switch. Remarkably, the fidelity is nearly independent on the 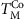

It is also worth discussing the role of the decoherence of the switch in the present schemes. As far as the CNOT gate scheme (compound **5**) is concerned, the contribution of the central Co switch to decoherence is similar to that of the rings, because the switch is temporarily excited to the *S*_*Z*_=+1/2 state during the CNOT gates. Conversely, only virtual excitations of the switch are involved in the implementation of the 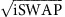 gate exploiting the redox-active linker. Hence, the fidelity remains very high even for dephasing time of the switch of the order of the gating time (some ns). This difference is shown by the two colour maps reported in Fig. 7. They show the dependence of the fidelity of a 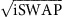 (left) and of a CNOT gate (right) on the dephasing times *T*_M_ of the rings and of the switch. It is evident that the fidelity of the 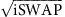 is practically independent on 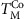 whereas its dependence on 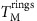 is much more pronounced. Conversely, the implementation of the CNOT on compound **5** leads to a similar dependence of the fidelity on 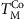 and 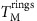.

The chemistry described above can be extended to make one-dimensional (1D) chains incorporating the two-Cr_7_Ni supramolecules, linked by either single cobalt sites or oxo-centred metal triangles. Such 1D chains have already been made involving single Cr_7_Ni units and [2]- and [3]-rotaxanes and the principles established, especially for 1D chains of rotaxanes^48^, should work well. The key steps are to include two functional groups per Cr_7_Ni ring, which has already been done for *iso-*nicotinate acid, and by functionalizing the termini of the central organic thread of rotaxanes. Inclusion of two different functionalities is more taxing but entirely feasible (see the detailed schemes in Supplementary Figs 30 and 31). Therefore the chemistry is potentially scalable.

While this challenging chemistry proceeds, it is possible to show that the schemes are theoretically scalable. The extension of a two-qubit QG to a multi-qubit register raises important issues concerning the propagation of errors. In such a register, we can identify two main sources of errors, whose effect increases with the number of qubits: pure dephasing and imperfect operation of the switch arising from a residual inter-qubit interaction still present in the ‘off' state.

Errors induced by pure dephasing (decoherence) increase with the overall computational time. However, a finite chain of qubits with interposed switches would allow simultaneous (parallel) manipulation of non-overlapping parts of the register, which drastically reduces the computation time, and hence decoherence-induced errors, with respect to a serial implementation. For instance, a setup based on compound **5** can be manipulated in parallel for interesting classes of quantum simulation algorithms^17^. This requires inclusion of two distinct switches in the structure—Switch_A_ and Switch_B_—with different responses to external stimuli, such that the 1D chain has as a repeat pattern -Qubit-Switch_A_-Qubit-Switch_B_-.

For compounds **7**, **8** and **9**, the scheme requires a local addressability of individual switches on the chain, which are separated by about 3 nm. For a first proof-of-principle experiment with a single assembly, an STM tip could be used to provide the best control. A parallel implementation of gates would be possible if different Co switches could be addressed individually at the same time. To achieve a scalable structure, a molecular chain of qubits might be layered onto a surface and addressed by means of a cross-bar architecture similar to that proposed in refs 16, 49. It is worth noting that Cr_7_Ni rings have already been deposited on surfaces, without significant modification of their magnetic properties^50^.

We now examine the scaling of the errors with the number of qubits in the register. As a first step, we consider the effect of pure dephasing on a set of non-interacting qubits subject to Lindblad (Markovian) dynamics. It can be shown (see ref. 51 and Supplementary Information) that the decoherence error *ɛ*=1−*F*^2^ on *N* qubits scales at most as 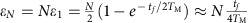, valid for *t*_*f*_*<<T*_M_ (where *t*_*f*_ is the optimal gating time). In the parallel quantum-simulator implementation considered in ref. 17, manipulating a chain of *N* qubits takes the same time as manipulating the shortest chain that contains the two distinct switches (qubit-Switch_A_-qubit-Switch_B_-qubit). Therefore *t*_*f*_ is limited by the value it assumes for a chain of three qubits, while in a serial scheme *t*_*f*_ increases linearly with *N*. By chemical engineering of Cr_7_Ni qubits, a *T*_M_ of 15 μs has been obtained^25^; this should allow the implementation of around twenty 2-qubit gate operations on a chain consisting of 10 qubits, while keeping the error below 0.1.

Finally, we analyse the consequences of an imperfect operation of the switch and their effect on scalability. For an ideal 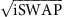 gate the inter-qubit exchange interaction would be completely turned off when the central node is in its diamagnetic state. Double electron–electron resonance measurements (Supplementary Fig. 27) reveal a very weak, residual interaction between the qubits. The resulting oscillations are on a time scale (ca. 0.2 μs) much longer than our gating times (a few ns) and could be corrected by means of refocusing techniques^29^. In the case of the CNOT scheme (compound **5**), the always ‘on' exchange interaction between the rings and the Co ion yields a small residual qubit–qubit coupling (Supplementary Methods). As a consequence, an unwanted slow evolution of the qubits occurs on a timescale *T*_UE_ of the order of a few hundreds of ns. Although this is longer than the CNOT gate time (about 30 ns), larger values of *T*_UE_ would be needed for performing sequences of many gates. This can be obtained by modifying the molecule to decrease the Co-ring exchange interaction. For instance, *T*_UE_ can be increased by a factor of 50 by reducing the Co-ring exchange interaction by a factor of three, which could be achieved chemically by adding an extra phenyl group between the ring and the central node. A similar reduction of the residual qubit–qubit coupling can be obtained by increasing the static magnetic field.

In summary, we have described two different schemes for universal quantum information processing, based on either local or global control of the qubit–qubit interaction. We have demonstrated that the flexibility of molecular {Cr_7_Ni} qubits makes them suitable for the implementation of each of these two schemes, if properly functionalized and linked by means of a supra-molecular design strategy. The two-qubit units can be controlled either magnetically or electrically, and implement either the CNOT or 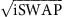 perfectly entangling gates. Our realistic simulations, based on experimental parameters and including decoherence, show that these gates can be performed with remarkably high fidelity. The modular strategy proposed here offers a degree of control in terms of the magnitude of the coupling between molecular spin qubits, the spatial orientation of the modules and the possibility to have a switchable interaction that represents a significant step forward with respect to previous achievements on the assembly of qubits.

Since future developments of quantum technology are unpredictable, we emphasize the importance of pursuing both these two parallel roads towards the actual realization of a quantum computer.

## Additional information

**Accession codes:** The X-ray crystallographic coordinates for structures reported in this Article have been deposited at the Cambridge Crystallographic Data Centre (CCDC), under deposition number CCDC 1029608–1029613 and 1415380–1415383. These data can be obtained free of charge from The Cambridge Crystallographic Data Centre via www.ccdc.cam.ac.uk/data_request/cif.

**How to cite this article:** Ferrando-Soria, J. *et al*. A modular design of molecular qubits to implement universal quantum gates. *Nat. Commun.* 7:11377 doi: 10.1038/ncomms11377 (2016).

## Supplementary Material

Supplementary Information Supplementary Figures 1-31, Supplementary Tables 1-12, Supplementary Methods and Supplementary References

Supplementary Data 1 Cif files for compounds **3** to **11**

## Figures and Tables

**Table 1:** Spin-Hamiltonian parameters used for simulation of the W-band EPR spectra of **5**.

	***g***_***x***_	***g***_***y***_	***g***_***z***_		***J***_***x***_ (**cm**^−1^)	***J***_***y***_ (**cm**^−1^)	***J***_***z***_ (**cm**^−1^)
{Cr_7_Ni-O_2_C-terpy}	1.78	1.78	1.74	*J*_12_	−0.14	0.34	0.17
{Cr_7_Ni-O_2_C-py}	1.74	1.78	1.78	*J*_23_	−0.07	0.17	0.34
Co(II)	1.78	4.25	6.50				

*J*_12_, *J*_23_ and *g*={*g*_*x*_*,g*_*y*_*,g*_*z*_} are diagonal tensors.
