# Supplementary material for: A modular design of molecular qubits to implement universal quantum gates
Source: Nat Commun. 2016 Apr 25;7:11377. doi: 10.1038/ncomms11377 (PMC4848482; doi:10.1038/ncomms11377)
Supplement: Supplementary Information — Supplementary Figures 1-31, Supplementary Tables 1-12, Supplementary Methods and Supplementary References [file ncomms11377-s1.pdf]

Supplementary Figures

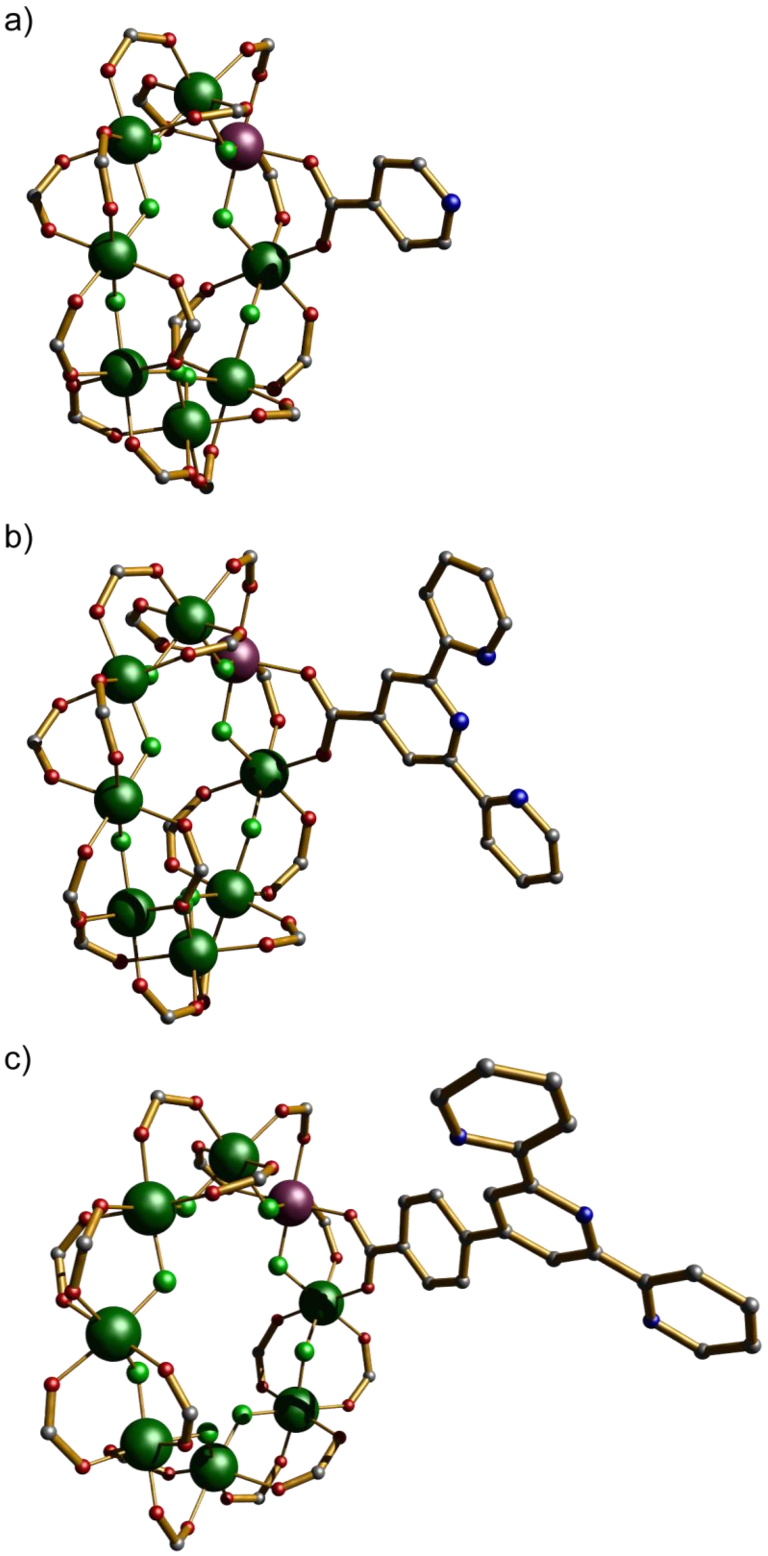

Supplementary Figure 1. a), b) and c) Crystal structure of **2**, **3** and **4**, respectively. Colour code: Cr, green; Ni, purple; N, cyan; O, red; C, grey; F, pale green. $^{n}Pr_2NH_2^+$ cations are not shown (H atoms and *tert*-butyl groups are omitted for clarity).

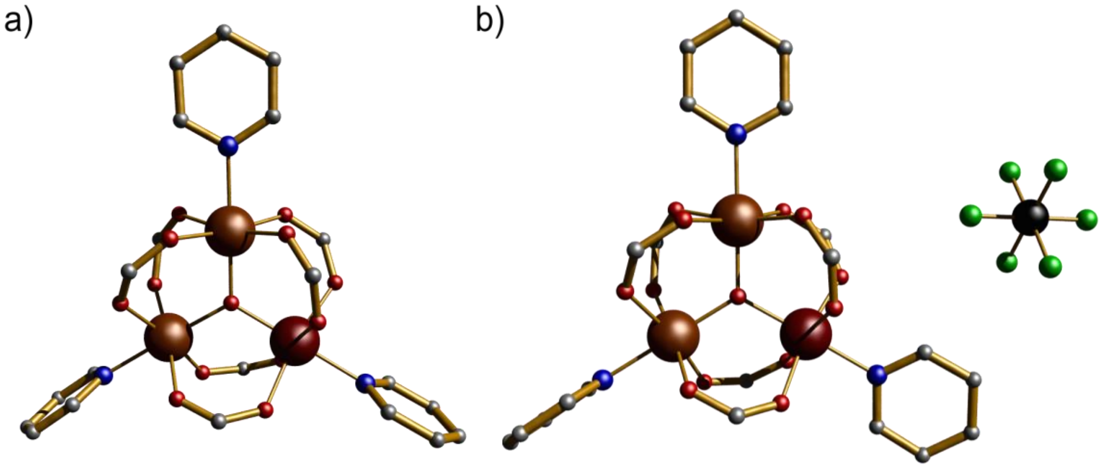

Supplementary Figure 2. a) and b) Crystal structure of **6** and **6**$^{\mathbf{ox}}$, respectively. Colour code: Co, dark red; Ru, brown; N, cyan; O, red; C, grey; P, black; F, pale green. H atoms and *tert*-butyl groups are omitted for clarity.

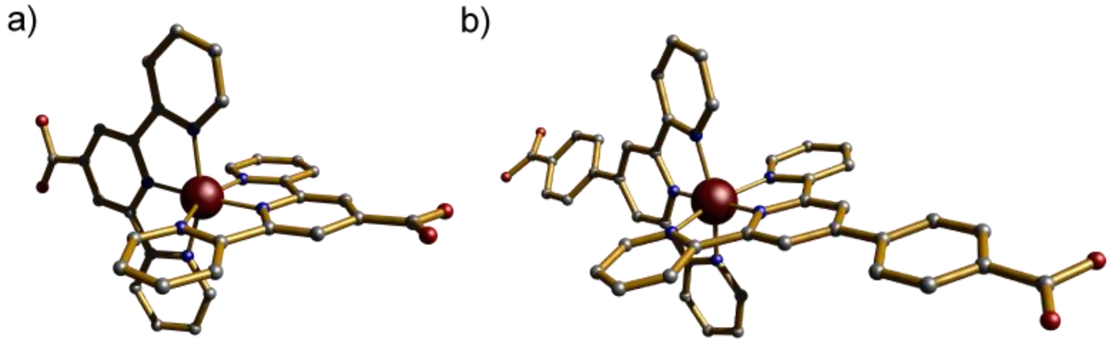

Supplementary Figure 3. a) and b) Crystal structure of **10** and **11**, respectively. Colour code: Co, dark red; N, cyan; O, red; C, grey. BF_4^- counteranions and H omitted for clarity.

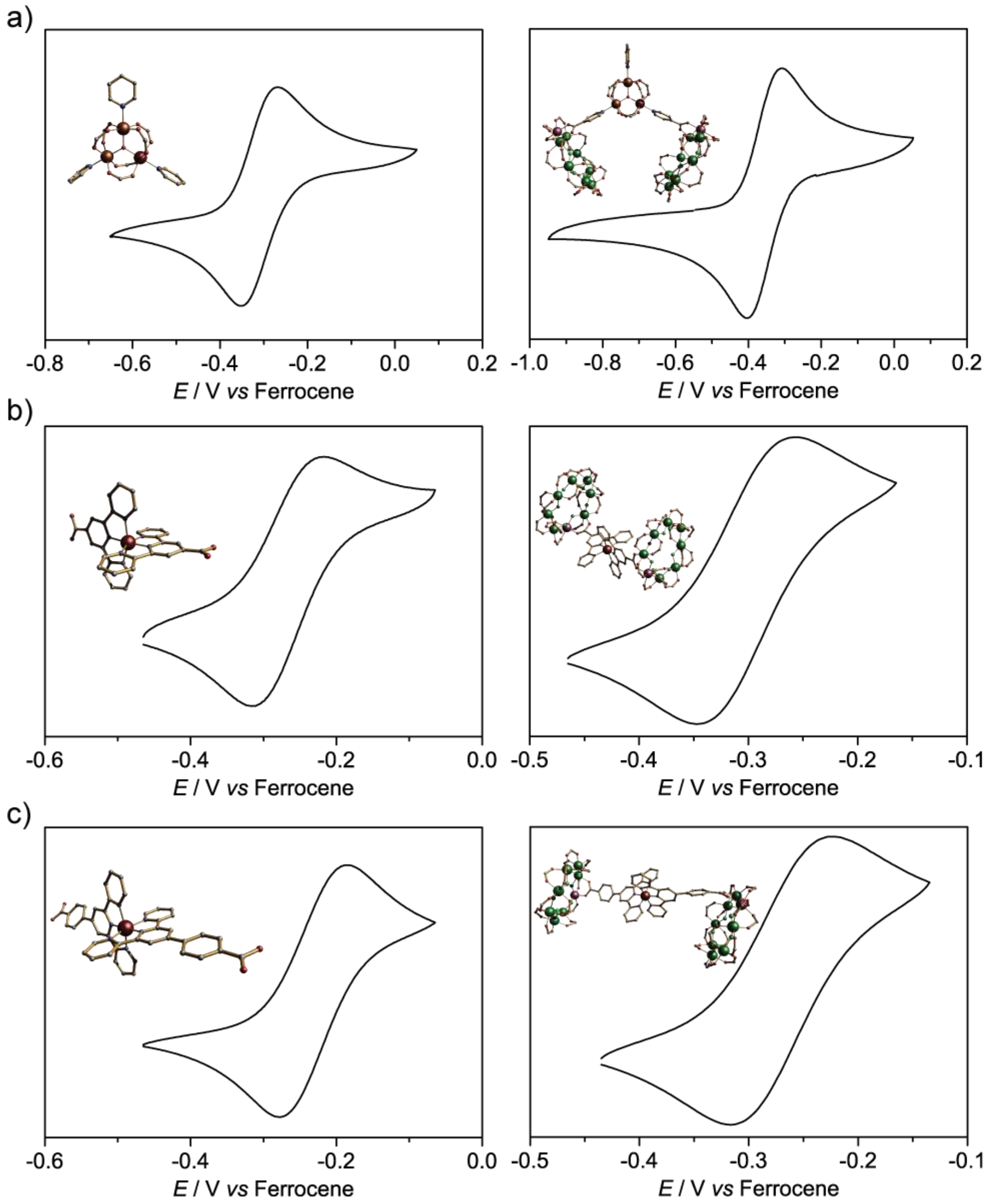

Supplementary Figure 4. a), b) and c) Cyclic voltammograms of the redox-switchable central nodes in isolation (left panel), and on the supramolecular two-qubits assemblies (right panel) of compounds **6**–**7**, **10–8b** and **11–9b**, respectively, in dichloromethane (0.1 M *n*NBu_4PF_6, 25 °C).

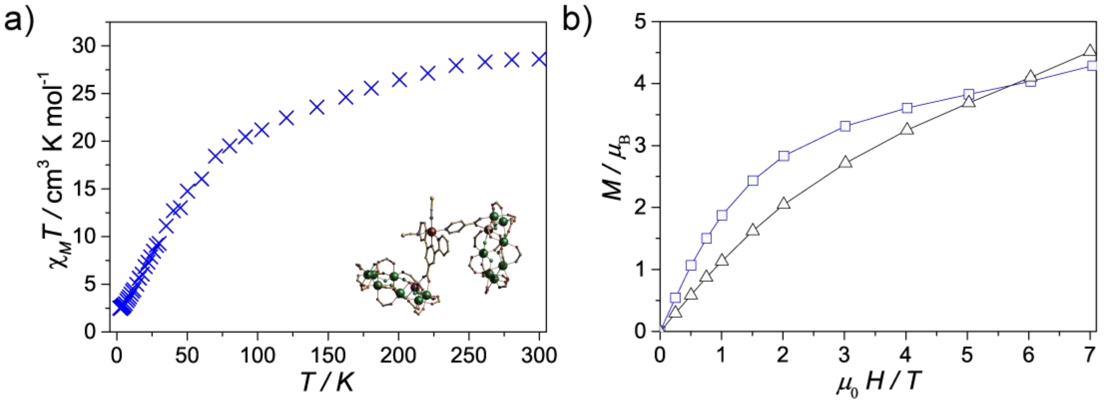

Supplementary Figure 5. a) Temperature dependence of $\chi_M T$ for **5** under an applied field of 1000 G. b) Field dependence of M/μ_β for compound **5** at 2 (blue trace) and 4 K (black trace).

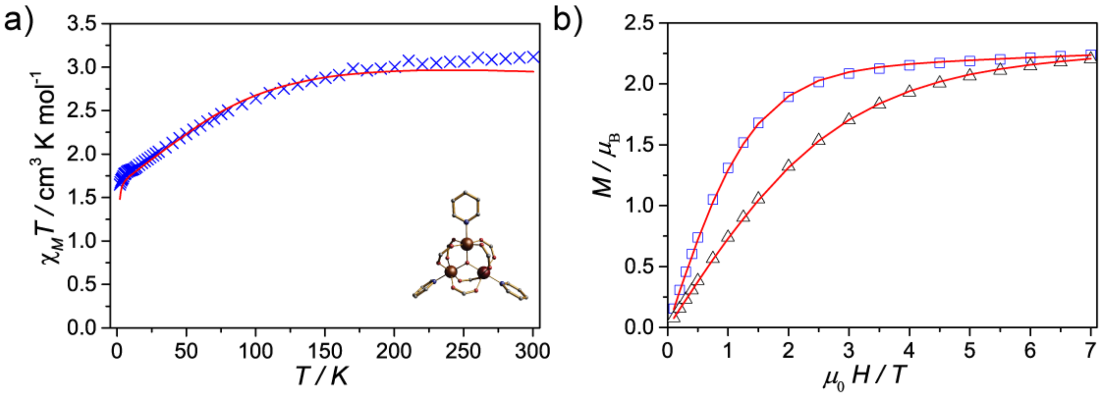

Supplementary Figure 6. a) Temperature dependence of $\chi_M T$ for **6** under an applied field of 5000 G. b) Field dependence of M/μ_β for compound **6** at 2 (blue squares) and 4 K (black triangles). The solid red lines correspond to the best-fit curves.

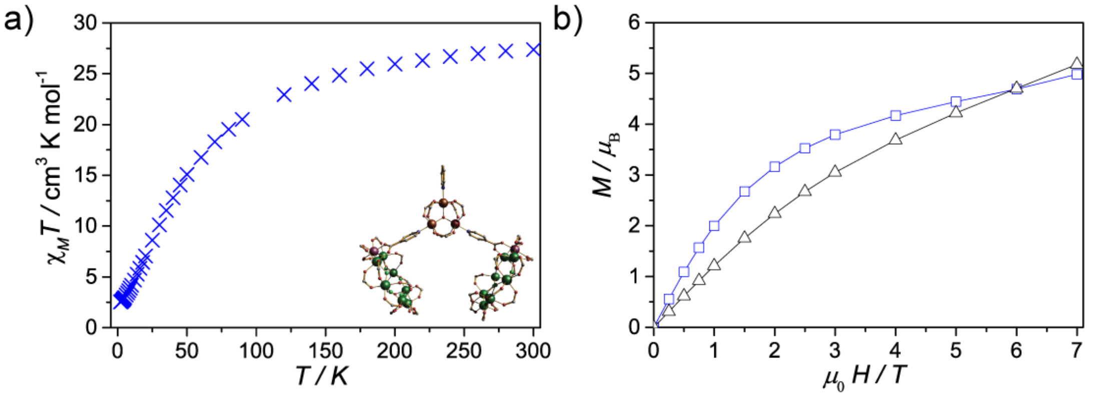

Supplementary Figure 7. a) Temperature dependence of $\chi_M T$ for **7** under an applied field of 1000 G. b) Field dependence of M/μ_β for compound **7** at 1.8 (blue trace) and 3 K (black trace).

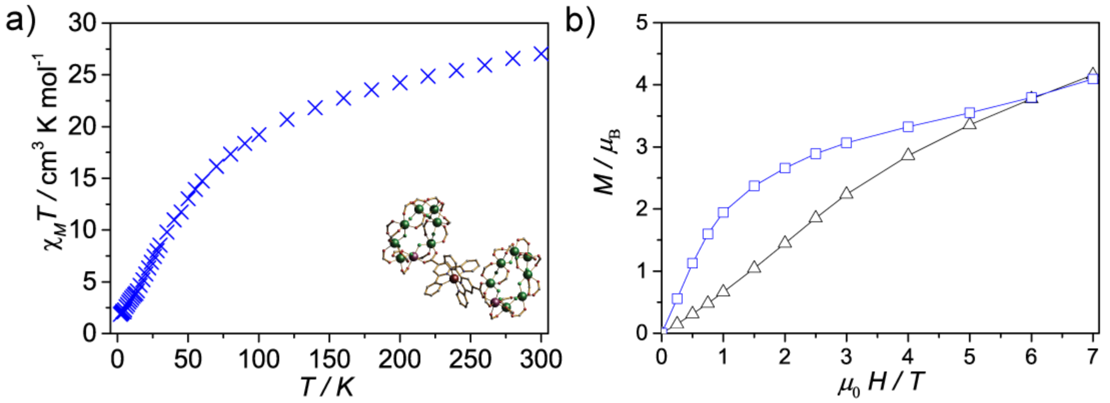

Supplementary Figure 8. a) Temperature dependence of $\chi_M T$ for **8b** under an applied field of 1000 G. b) Field dependence of M/μ_β for compound **8b** at 1.8 (blue trace) and 3 K (black trace).

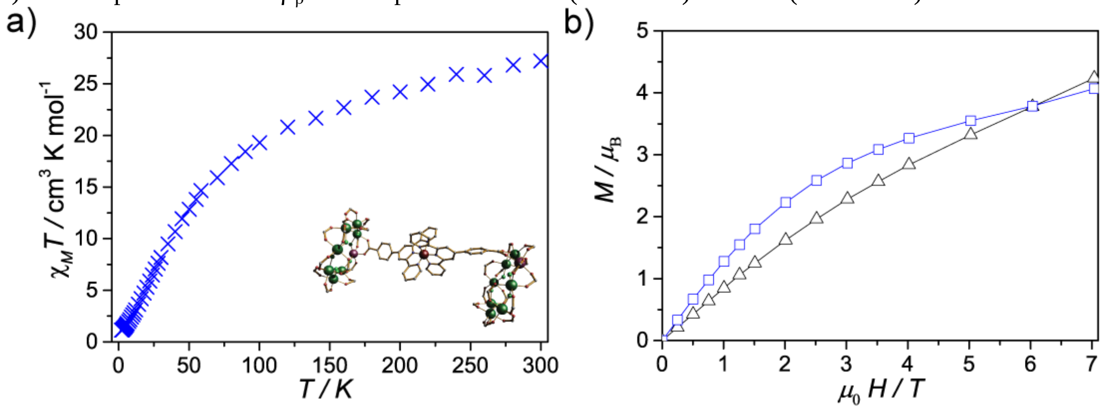

Supplementary Figure 9. a) Temperature dependence of $\chi_M T$ for **9b** under an applied field of 1000 G. b) Field dependence of M/μ_β for compound **9b** at 1.8 (blue trace) and 3 K (black trace).

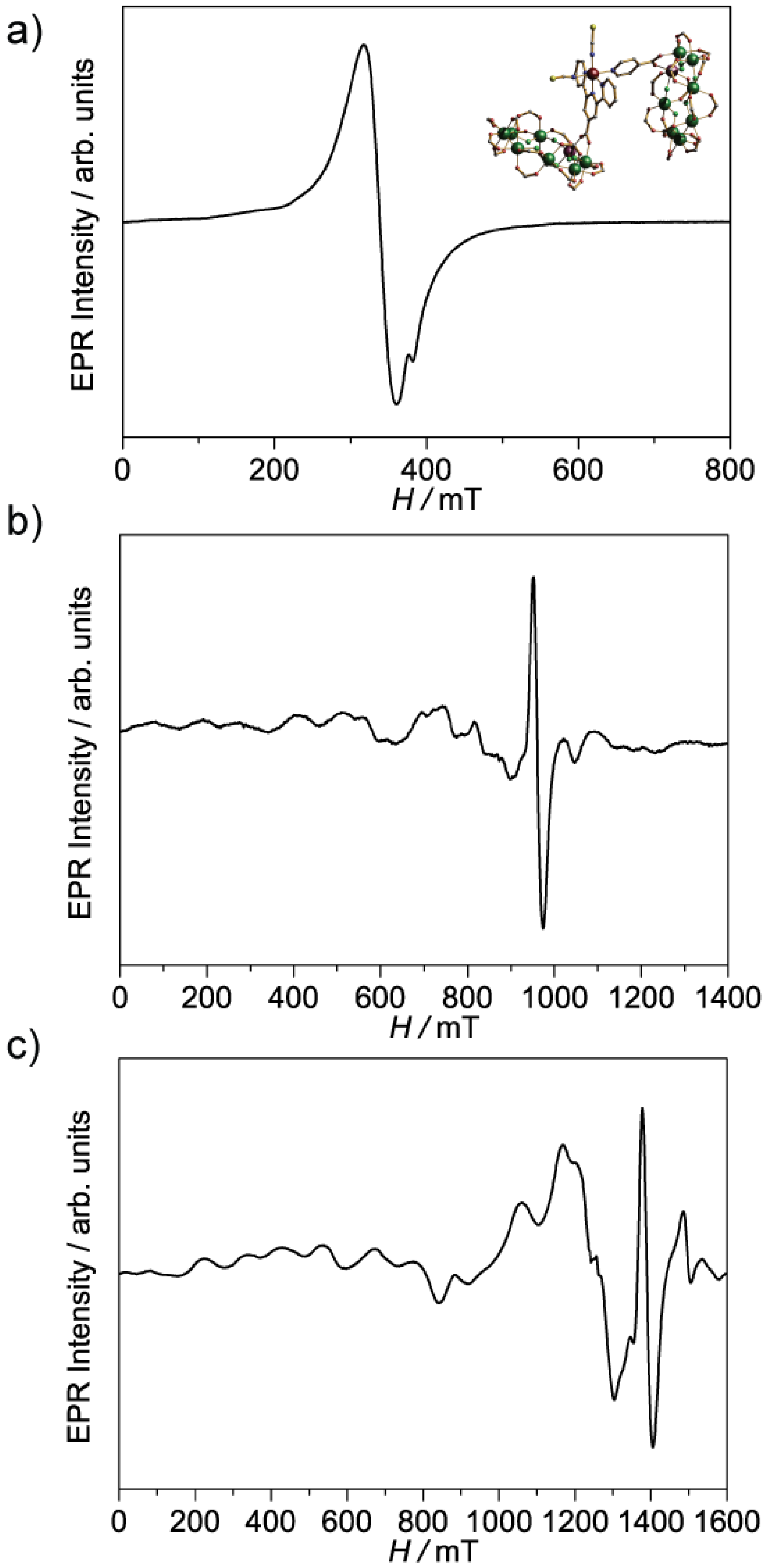

Supplementary Figure 10. a), b) and c) X-, K- and Q-band continuous wave electron paramagnetic resonance on powder of **5** at 5K, respectively.

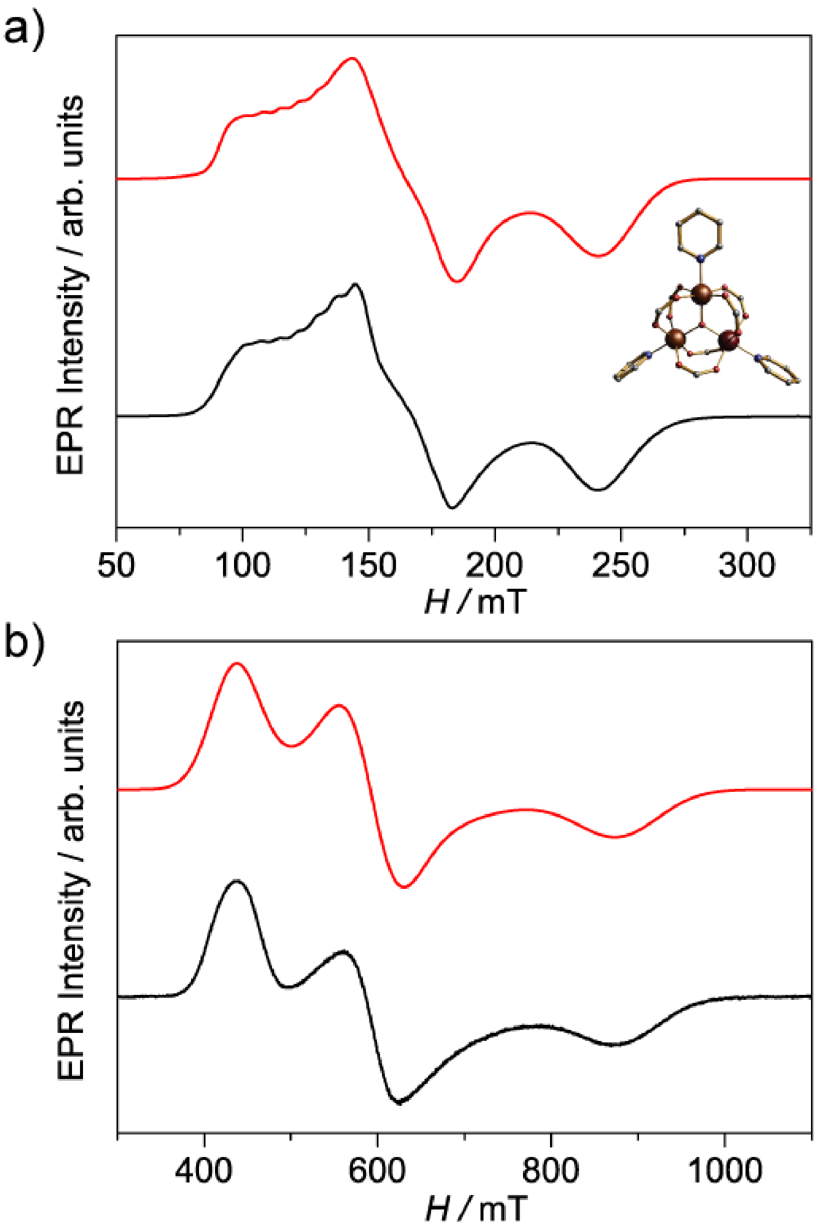

Supplementary Figure 11. a) Measured frozen solution X- band continuous wave electron paramagnetic resonance of **6** (black trace) and simulation frozen solution spectrum of **6** (red trace). b) Measured frozen solution Q- band continuous wave electron paramagnetic resonance of **6** (black trace) and simulation frozen solution spectrum of **6** (red trace). Simulation parameters: S_{eff} = $^{1}/_{2}$; g_x = 5.61; g_y = 4.05; g_z = 2.77; A_{xx} = 540; A_{yy} = 414; A_{zz} = 60 MHz.

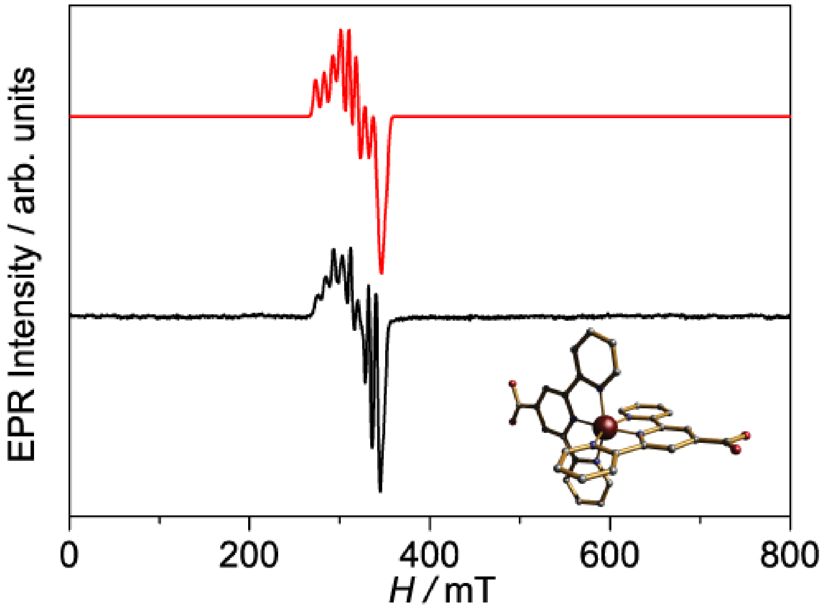

Supplementary Figure 12. Measured frozen solution X-band EPR spectra of **10** (black trace) and simulation frozen solution spectrum of **10** (red trace). Simulation parameters: S = $^{1}/_{2}$; g_x = 2.047; g_y = 2.076; g_z = 2.195; A_{xx} = 48; A_{yy} = 201; A_{zz} = 279 MHz.

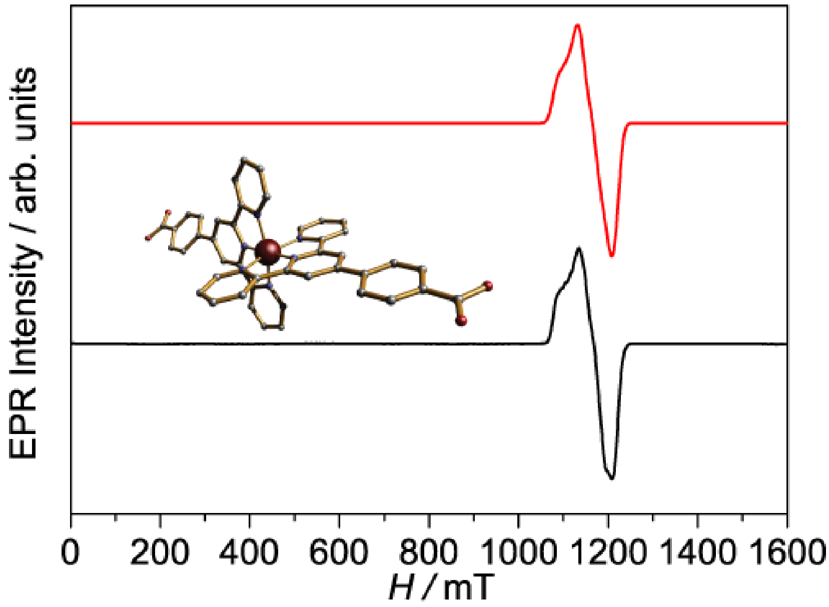

Supplementary Figure 13. Experimental (black trace) and simulated (red trace) powder Q-band specta of **11**. Simulation parameters: S = $^{1}/_{2}$; g_x = 2.022; g_y = 2.111; g_z = 2.215; A_{xx} = 14; A_{yy} = 183; A_{zz} = 278 MHz.

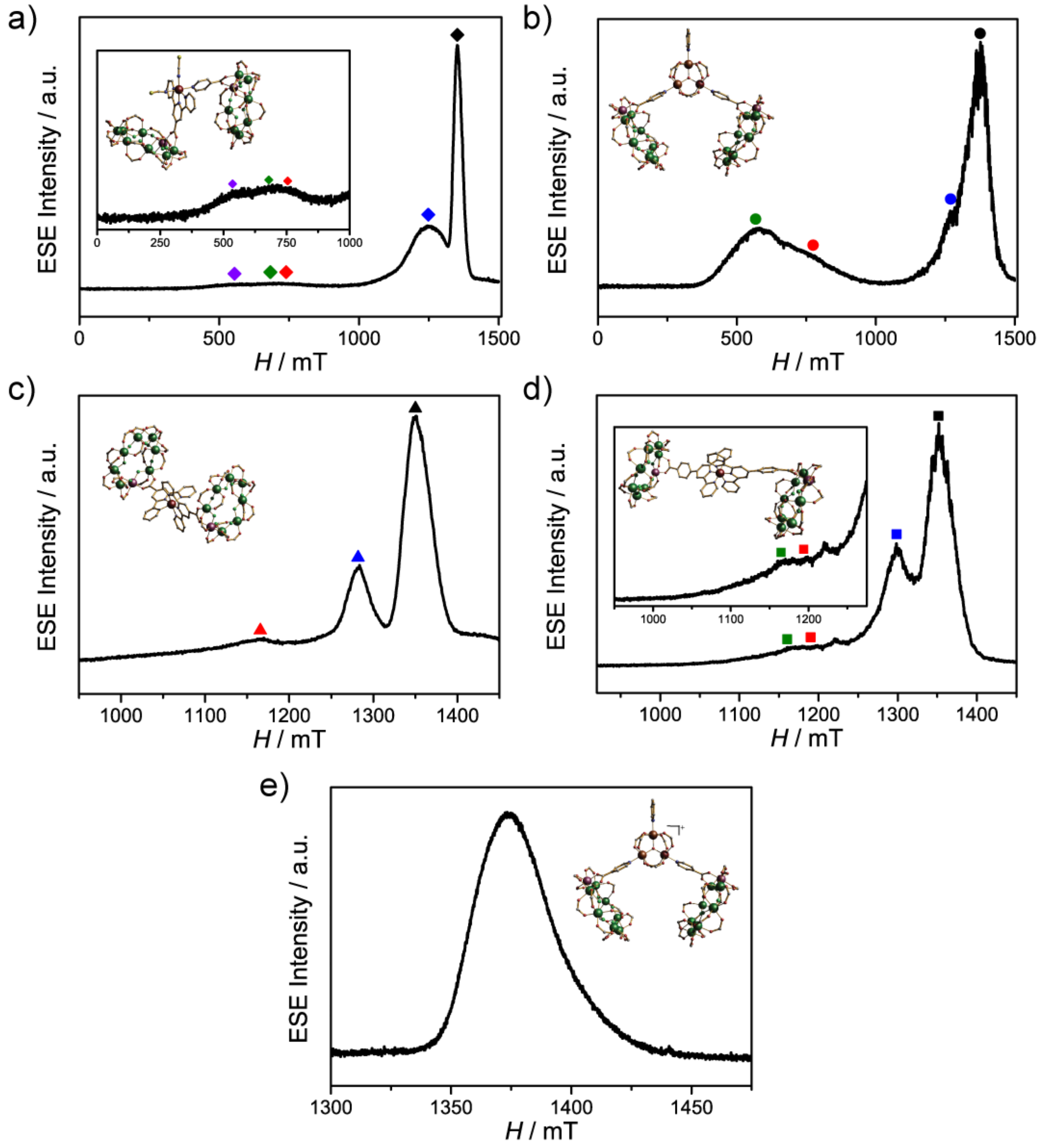

Supplementary Figure 14. Echo-detected field-swept spectra at Q-band for a) **5**, b) **7**, c) **8b**, d) **9b** and e) $\mathbf{7^{ox}}$ in 0.002 M (**5**, **7**, **8b** and **9b**) or 0.0001 M ($\mathbf{7^{ox}}$) toluene solutions, recorded with a primary echo sequence $\pi/2$-τ-π-τ-*echo* with π = 60 ns and τ = 300 ns.

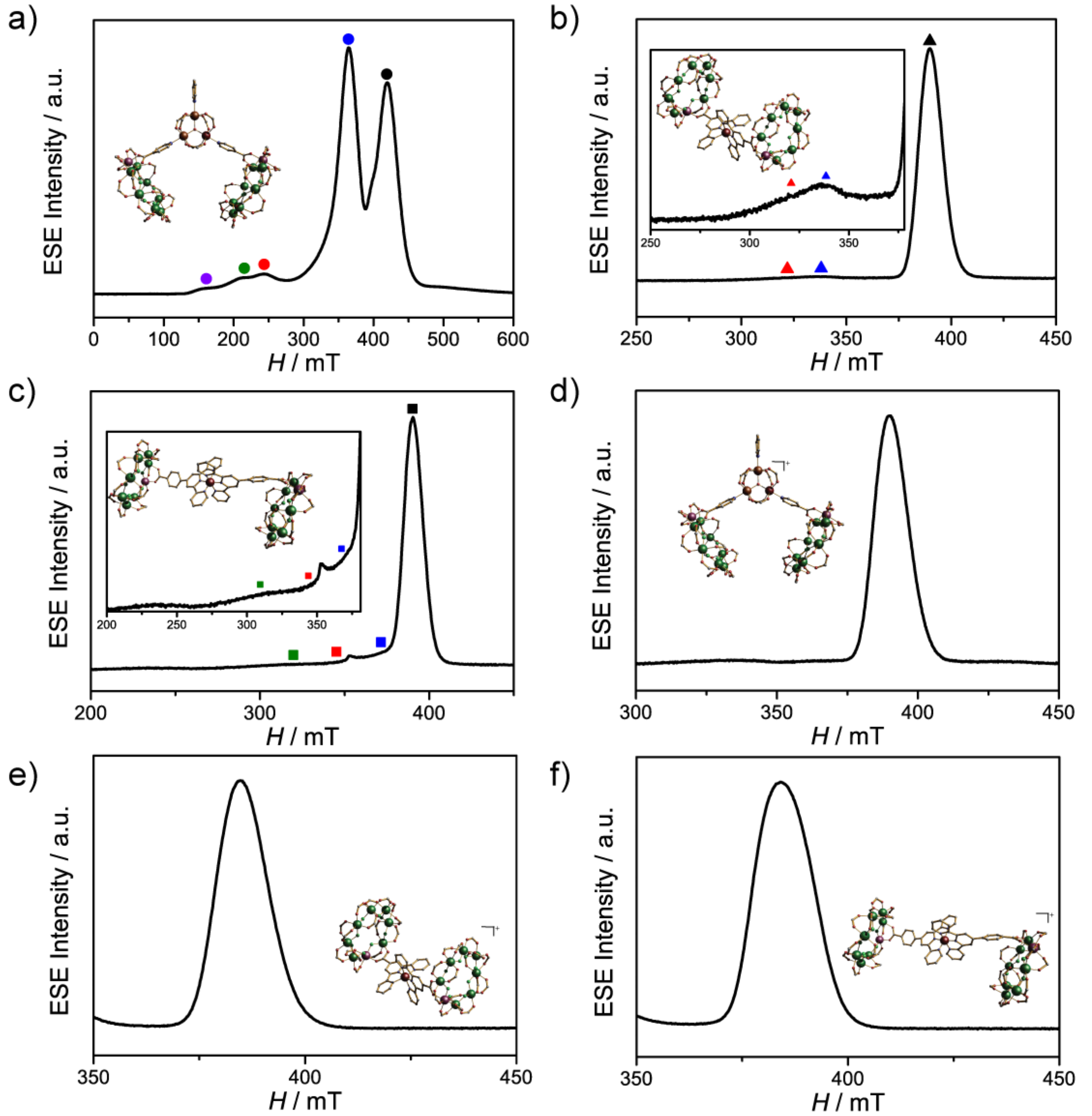

Supplementary Figure 15. Echo-detected field-swept spectra at X-band for a) **7**, b) **8b**, c) **9b**, d) **7^{ox}**, e) **8b^{ox}** and f) **9b^{ox}** in 0.002 M (**7**, **8b** and **9b**) or 0.0001 M (**7^{ox}**, **8b^{ox}** and **9b^{ox}**) toluene solutions, recorded with a primary echo sequence π/2-τ-π-τ-*echo* with π = 32 ns and τ = 300 ns.

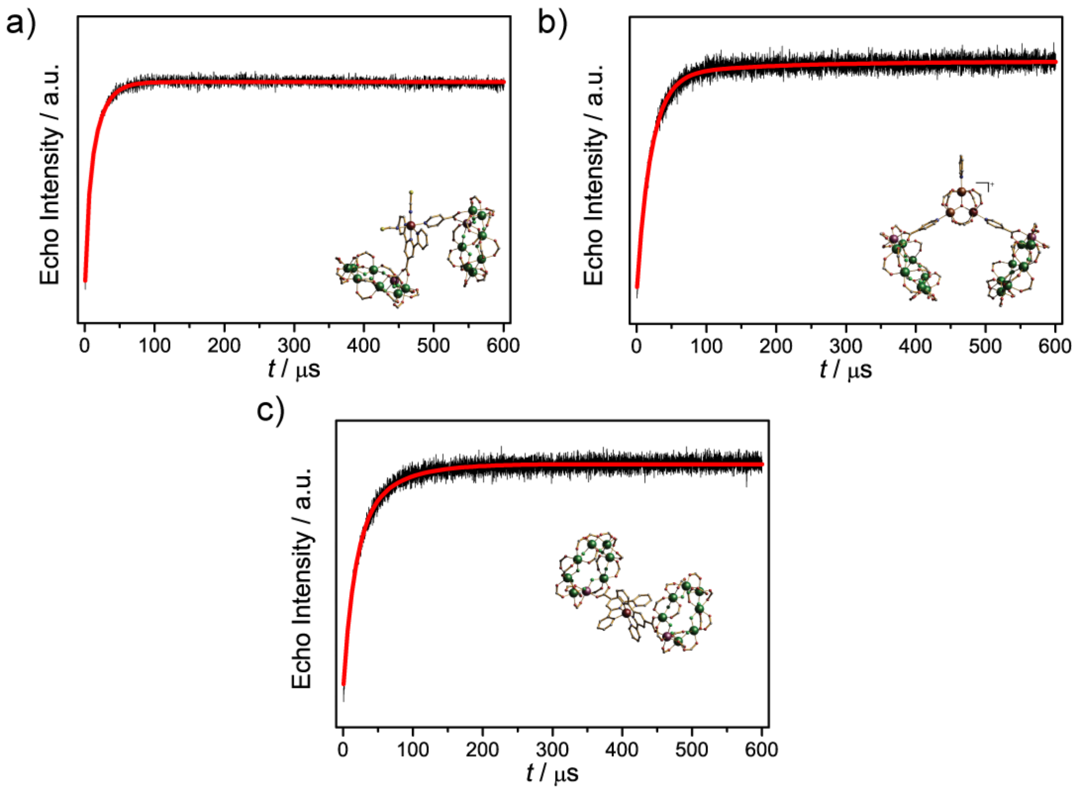

Supplementary Figure 16. Electron-spin-echo (ESE) inversion recovery curves at Q-band for a) **5**, b) **7^{ox}** and c) **8b** in diluted (0.0001 M) toluene solutions at the maximum resonance field for the ring in each compound. The red line is a fit to the exponential decay function $I(\mathrm{t}) = I_1 \exp(-t/T_1) + I_{SD} \exp(-t/T_{SD})$ from which the time constants T_1 and T_{SD} were deducted (see Table S4).

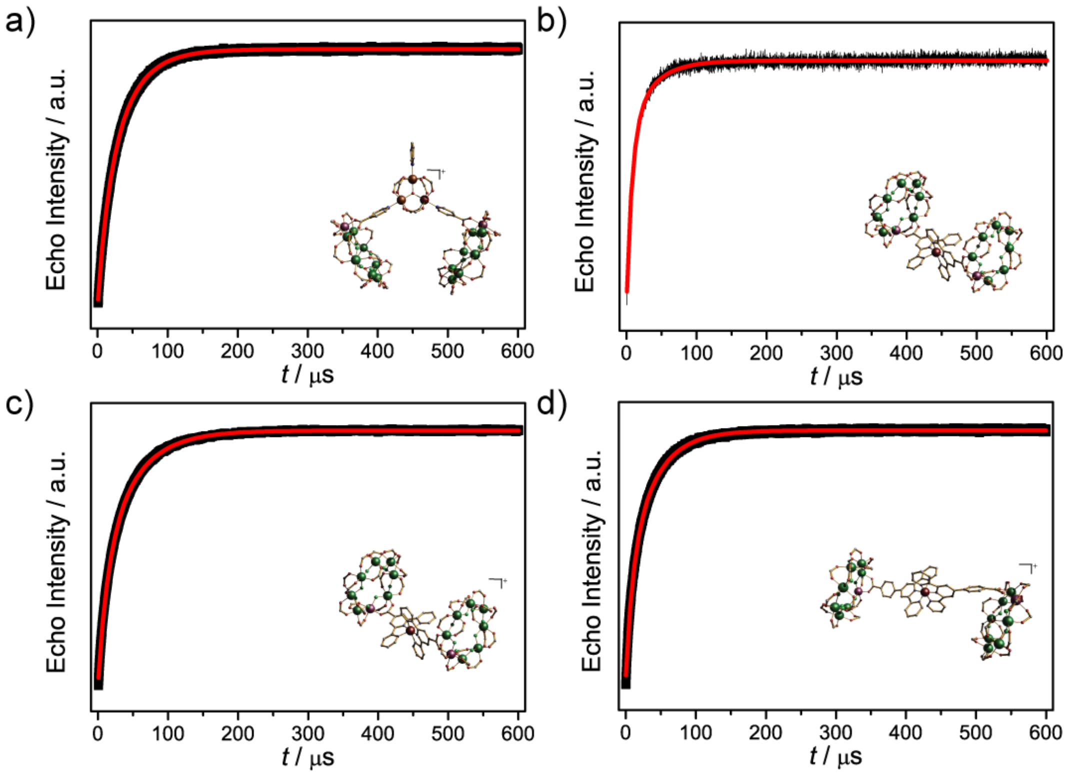

Supplementary Figure 17. Electron-spin-echo (ESE) inversion recovery curves at X-band for a) **7^{ox}**, b) **8b**, c) **8b^{ox}** and d) **9b^{ox}** in diluted (0.0001 M) toluene solutions at the maximum resonance field for the ring in each compound. The red line is a fit to the exponential decay function $I(\mathrm{t}) = I_1 \exp(-t/T_1) + I_{SD} \exp(-t/T_{SD})$ from which the time constants T_1 and T_{SD} were deducted (see Table S9).

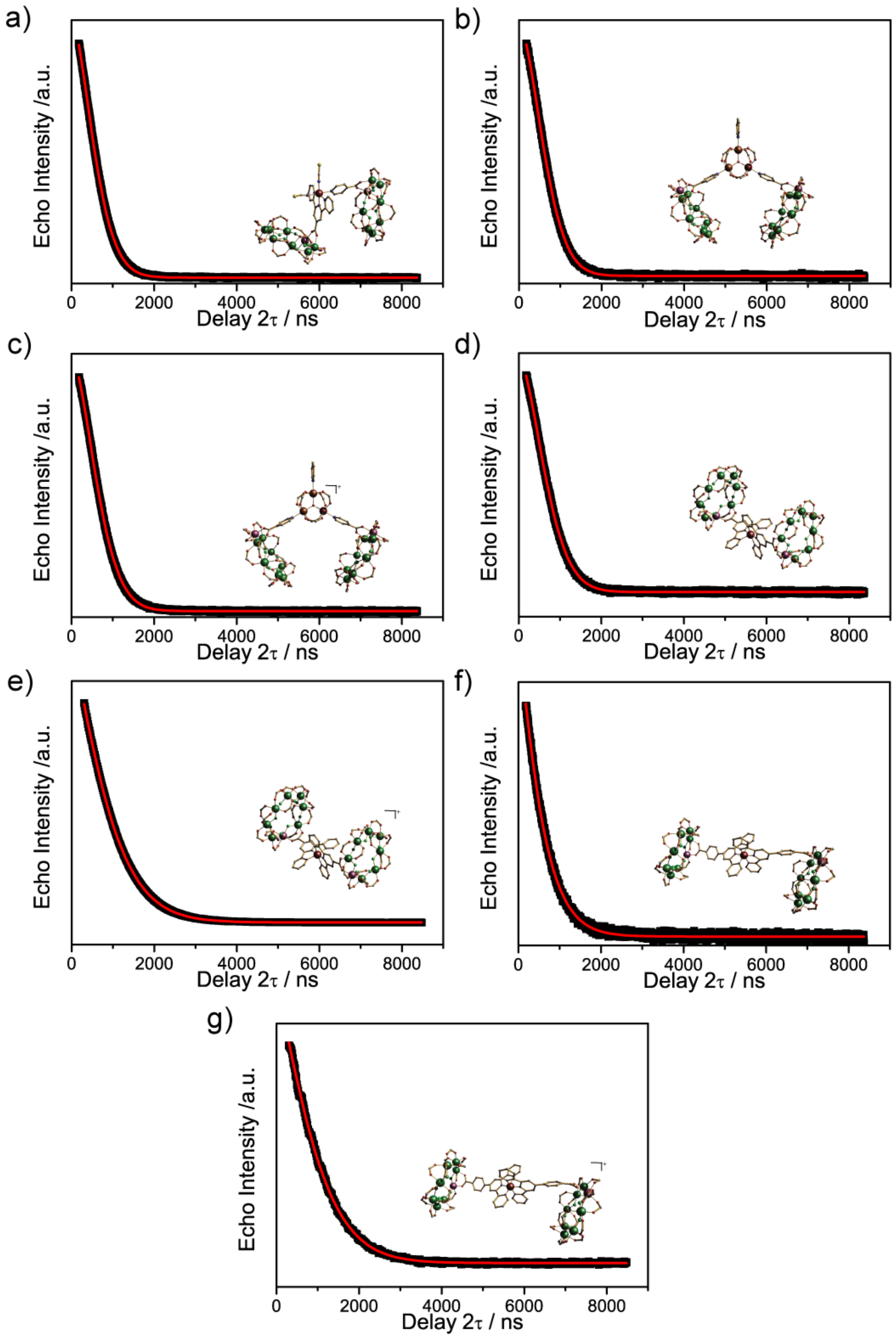

Supplementary Figure 18. Two pulse electron spin-echo decays at Q-band for a) **5**, b) **7**, c) $\mathbf{7^{ox}}$, d) **8b**, e) $\mathbf{8b^{ox}}$, e) **9b** and f) $\mathbf{9b^{ox}}$ in diluted (0.0001 M) toluene solutions at the maximum resonance field for the ring in each compound, recorded with pulses of π = 128 ns. The red lines represent fits to Eq. 2, $I(2\tau)=I(0)\exp[(-2\tau/T_M)^s]$, with T_M and s values listed in Table S4**.**

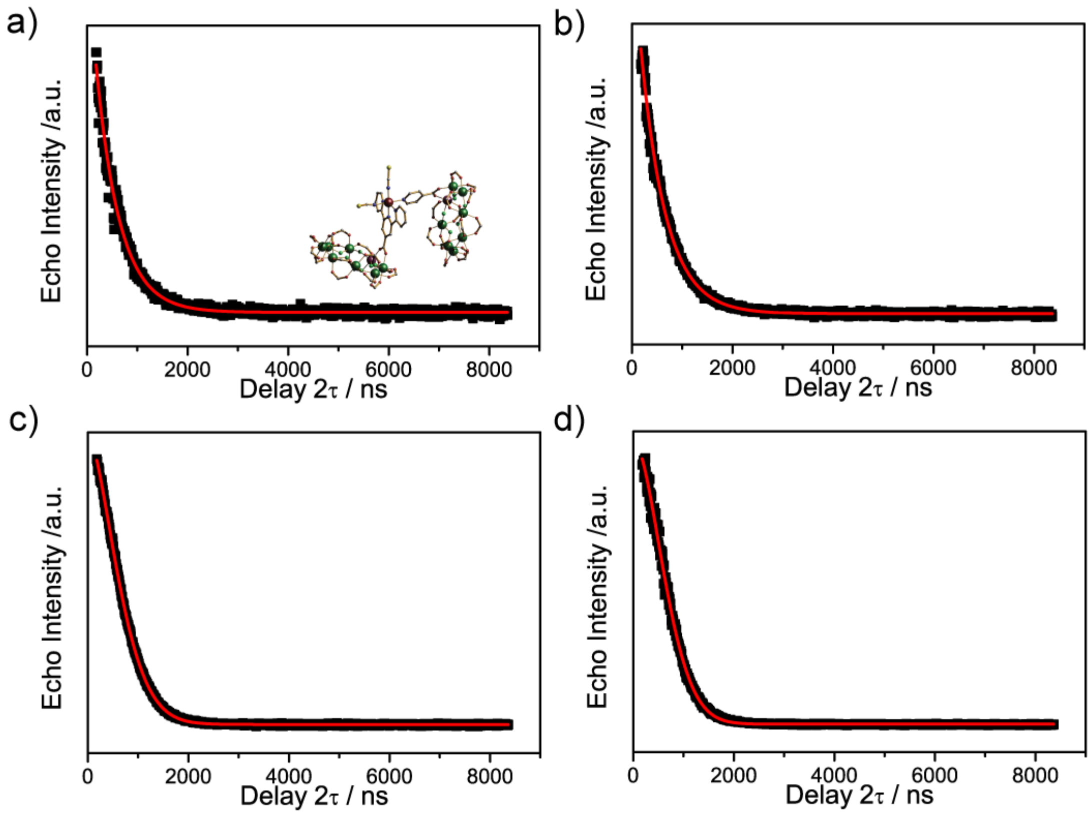

Supplementary Figure 19. Two pulse electron spin-echo decays at Q-band for **5** in toluene solutions (0.002 M) at some representative resonance fields a) 556, b) 709, c) 1247 and d) 1350 mT, recorded with pulses of π = 128 ns. The red lines represent fits to mono-exponential decay function (for the central node resonances) or to $I(2\tau)=I(0)\exp[(-2\tau/T_M)^s]$ (for the ring resonances), with T_M and s values listed in Table S5**.**

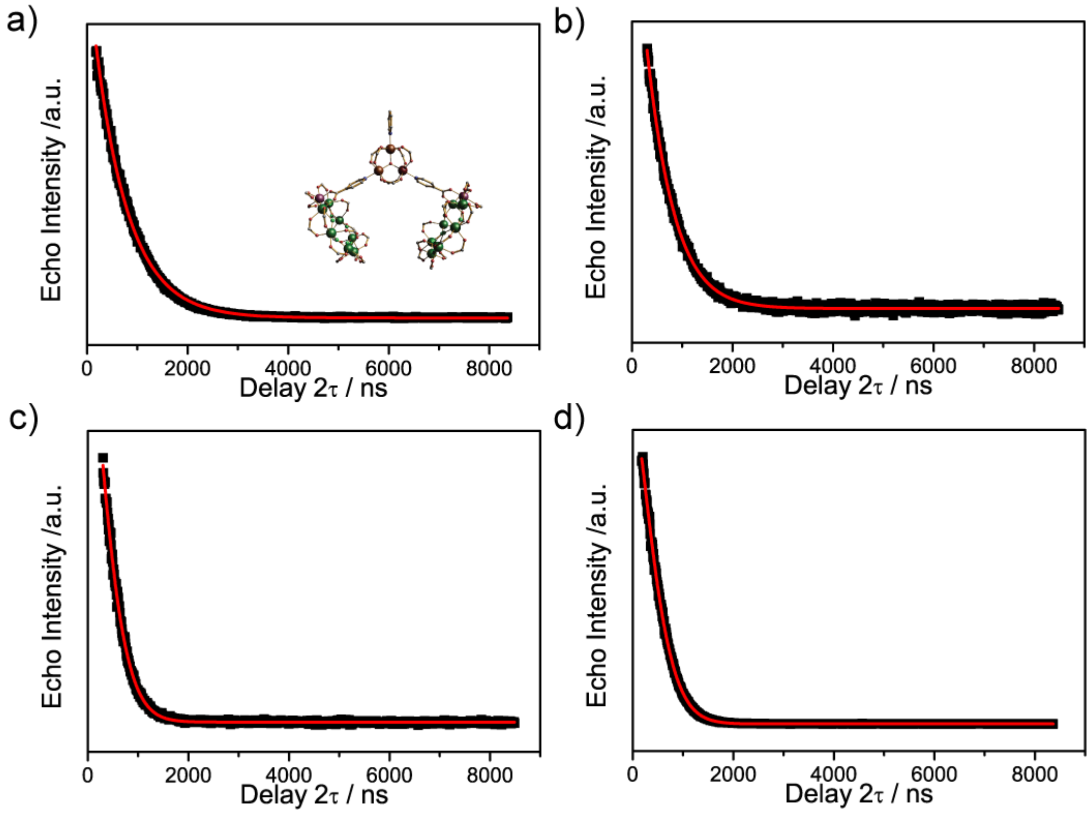

Supplementary Figure 20. Two pulse electron spin-echo decays at Q-band for **7** in toluene solutions (0.002 M) at some representative resonance fields a) 557, b) 776, c) 1260 and d) 1350 mT, recorded with pulses of π = 128 ns. The red lines represent fits to mono-exponential decay function (for the central node resonances) or to $I(2\tau)=I(0)\exp[(-2\tau/T_M)^s]$ (for the ring resonances), with T_M and s values listed in Table S6**.**

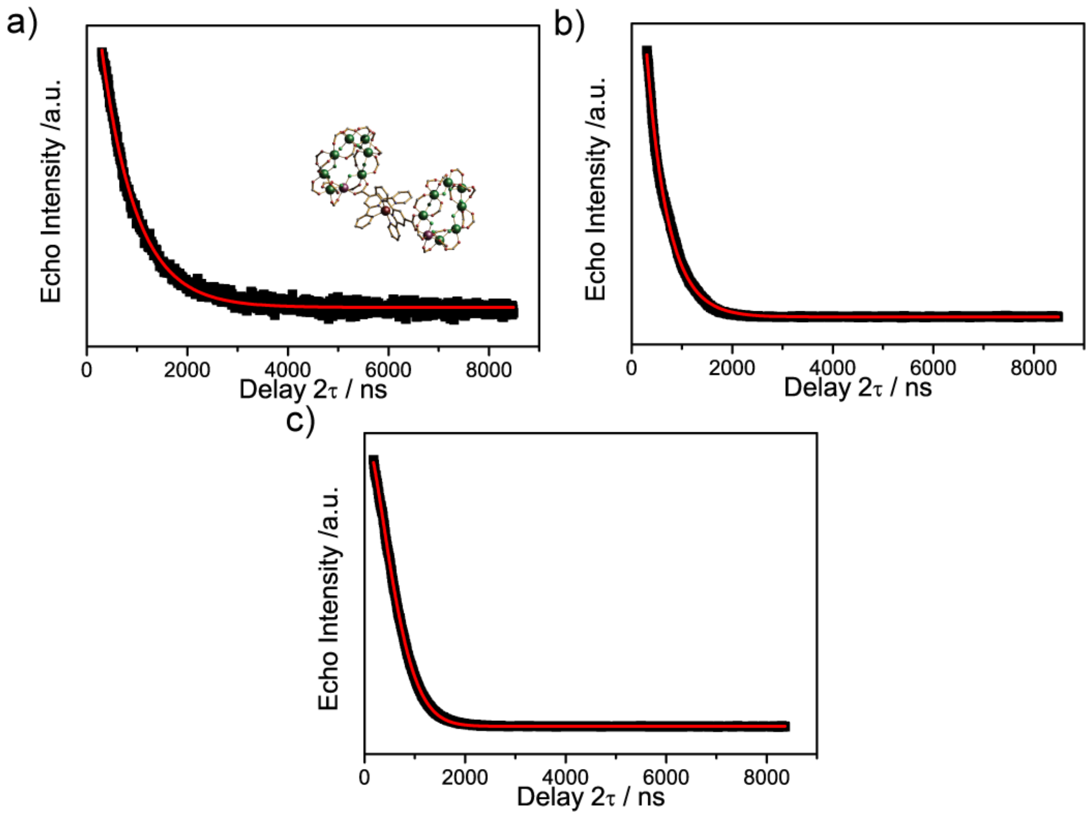

Supplementary Figure 21. Two pulse electron spin-echo decays at Q-band for **8b** in toluene solutions (0.002 M) at some representative resonance fields a) 1166, b) 1281 and c) 1350 mT, recorded with pulses of π = 128 ns. The red lines represent fits to mono-exponential decay function (for the central node resonances) or to $I(2\tau)=I(0)\exp[(-2\tau/T_M)^s]$ (for the ring resonances), with T_M and s values listed in Table S7**.**

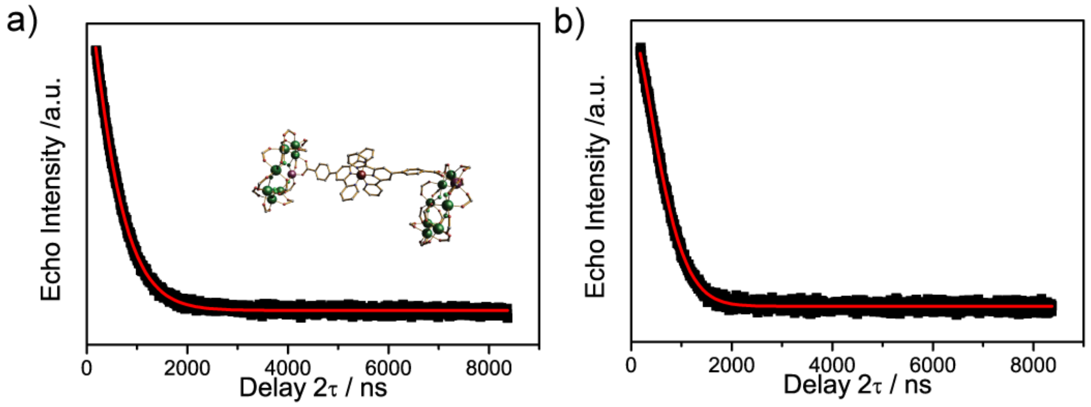

Supplementary Figure 22. Two pulse electron spin-echo decays at Q-band for **9b** in toluene solutions (0.002 M) at some representative resonance fields a) 1188 and b) 1307 mT, recorded with pulses of π = 128 ns. The red lines represent fits to mono-exponential decay function (for the central node resonances) or to $I(2\tau)=I(0)\exp[(-2\tau/T_M)^s]$ (for the ring resonances), with T_M and s values listed in Table S8**.**

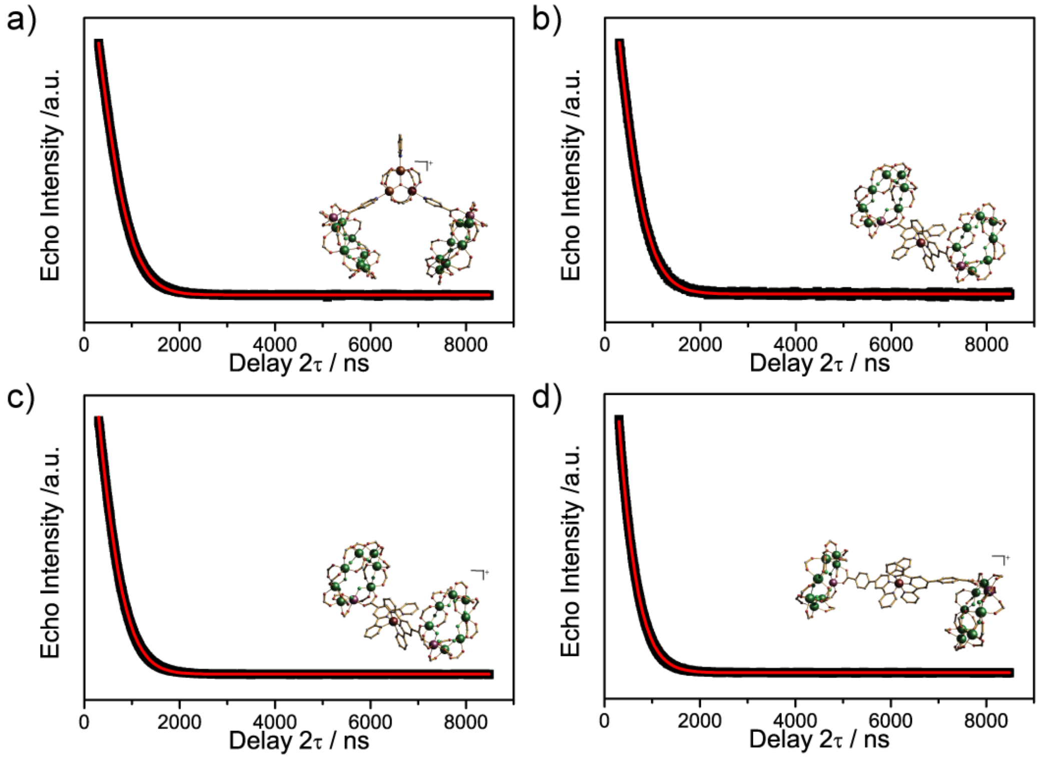

Supplementary Figure 23. Two pulse electron spin-echo decays at X-band for a) $\mathbf{7^{ox}}$, b) **8b**, c) $\mathbf{8b^{ox}}$ and d) $\mathbf{9b^{ox}}$ in diluted (0.0001 M) toluene solutions at the maximum resonance field for the ring in each compound, recorded with pulses of π = 128 ns. The red lines represent fits to Eq. 2, $I(2\tau)=I(0)\exp[(-2\tau/T_M)^s]$, with T_M and s values listed in Table S9.

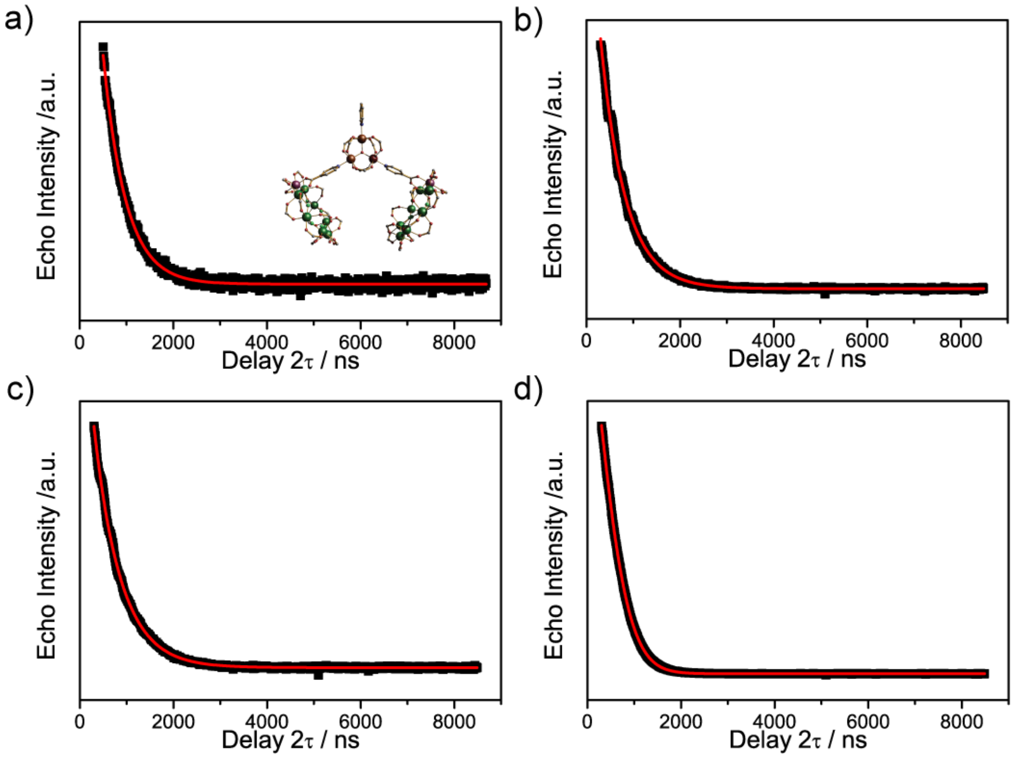

Supplementary Figure 24. Two pulse electron spin-echo decays at X-band for **7** in toluene solutions (0.002 M) at some representative resonance fields a) 161, b) 214, c) 244 and d) 420 mT, recorded with pulses of π = 128 ns. The red lines represent fits to mono-exponential decay function (for the central node resonances) or to $I(2\tau)=I(0)\exp[(-2\tau/T_M)^s]$ (for the ring resonances), with T_M and s values listed in Table S10**.**

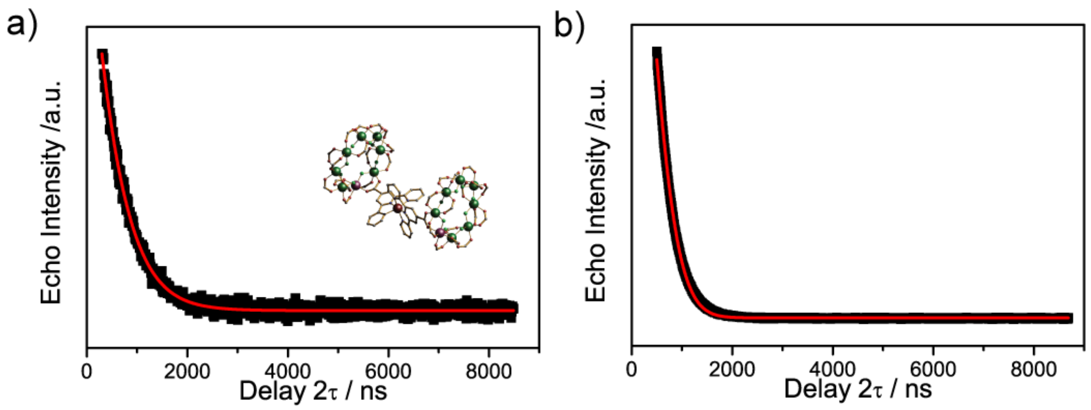

Supplementary Figure 25. Two pulse electron spin-echo decays at X-band for **8b** in toluene solutions (0.002 M) at some representative resonance fields a) 322 and b) 390 mT, recorded with pulses of π = 128 ns. The red lines represent fits to mono-exponential decay function (for the central node resonances) or to $I(2\tau)=I(0)\exp[(-2\tau/T_M)^s]$ (for the ring resonances), with T_M and s values listed in Table S11**.**

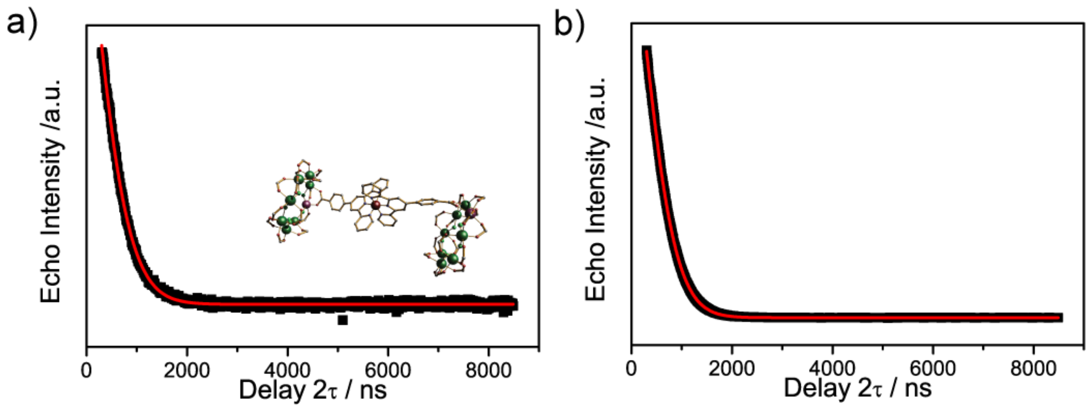

Supplementary Figure 26. Two pulse electron spin-echo decays at X-band for **9b** in toluene solutions (0.002 M) at some representative resonance fields a) 341 and b) 370 mT, recorded with pulses of π = 128 ns. The red lines represent fits to mono-exponential decay function (for the central node resonances) or to $I(2\tau)=I(0)\exp[(-2\tau/T_M)^s]$ (for the ring resonances), with T_M and s values listed in Table S12**.**

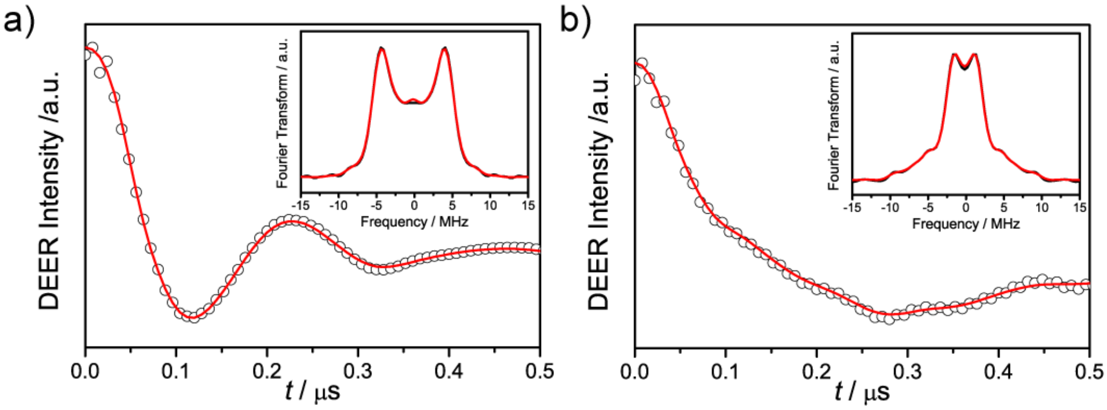

Supplementary Figure 27. DEER data at X-band for compounds $\mathbf{7}^{ox}$ (left) and $\mathbf{9b}^{ox}$ (right) in diluted (0.0001 M) toluene solutions at 2.5 K, recorded at the maximum resonance field for the ring in each compound with detection and pump frequencies of ν_1 = 9.7648GHz and ν_2 = ν_1-90MHz respectively. The inset represents the Fourier transform of the DEER signal (experimental: black; calculation: red).

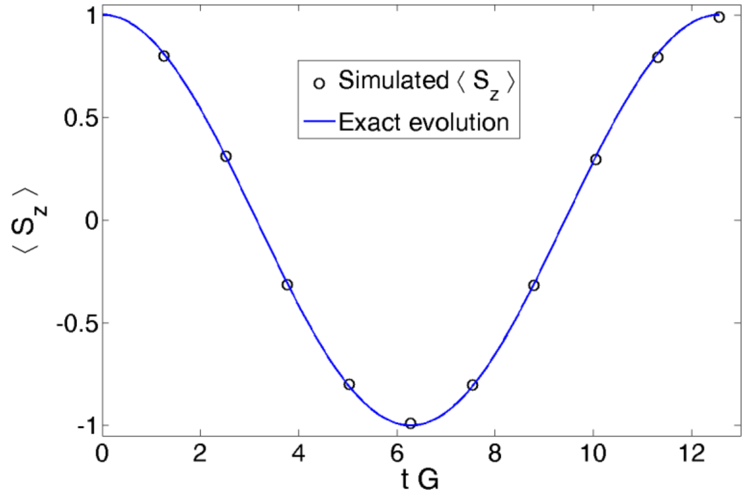

Supplementary Figure 28. Quantum simulation of an antisymmetric Hamiltonian. Oscillation of the expectation value of the magnetization in the quantum simulation of the antisymmetric Hamiltonian $H_a = G(S_1^z S_2^x - S_1^x S_2^z)$. The simulation (points) is performed with the parameters obtained from EPR for **5** and is in excellent agreement with the exact evolution (continuous line). The time required for the whole simulation is about 70 ns (much shorter than the qubit dephasing time) and the average fidelity is 98.5 %. The intensity of the oscillating field at the pulse maximum is 50 G, and we assume a static field is 5 T directed along *z*.

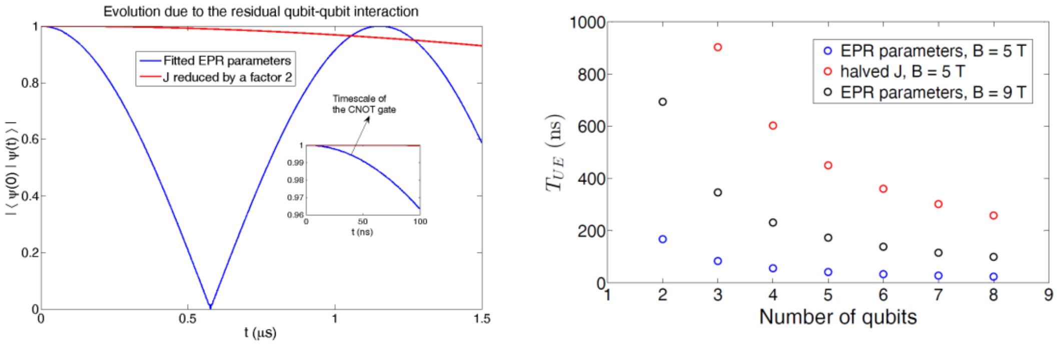

Supplementary Figure 29. Left: Fidelity of a 2-qubit state subject to an idle evolution (compound **5**) as a function of time (blue curve, with parameters fitted from EPR). The evolution is induced by the residual qubit-qubit coupling. **Inset:** zoom on the timescale of the CNOT gate. The red line reports the evolution calculated with halved *J* constants. **Right:** unwanted evolution time (T_{UE}) against number of qubits in a one-dimensional chain. T_{UE} is defined as the time required to reduce the fidelity to 0.9. Different sets of points compare the effect of a reduction of the exchange constants or of an increase of the magnetic field. The *N*-qubit state was initialized in $\frac{|0101...\rangle + |1010...\rangle}{\sqrt{2}}$, which was checked to be one of the most error-prone.

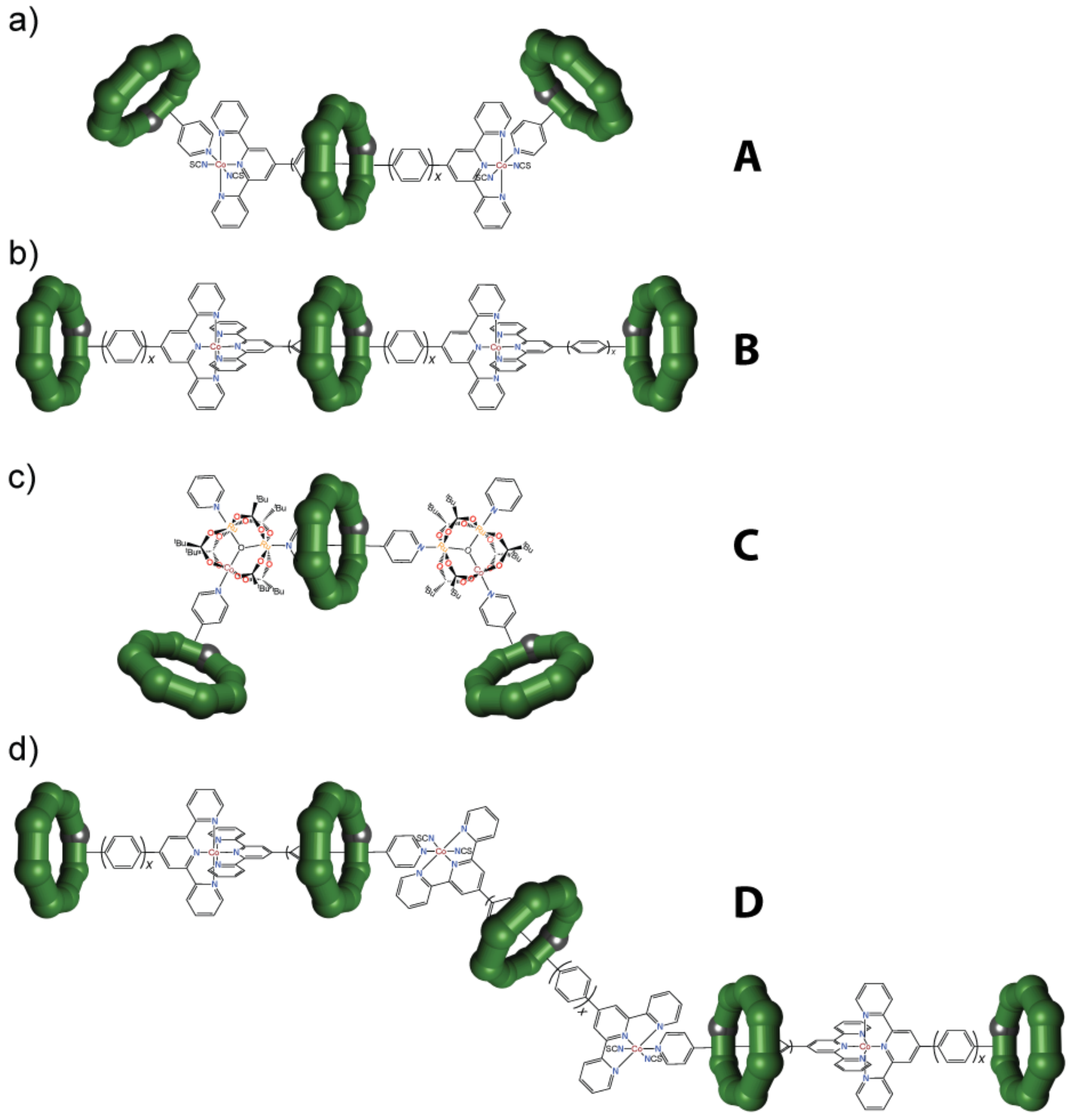

Supplementary Figure 30. Schematic representation of some potential prototypes of different sizes of finite chains made up of molecular qubits.

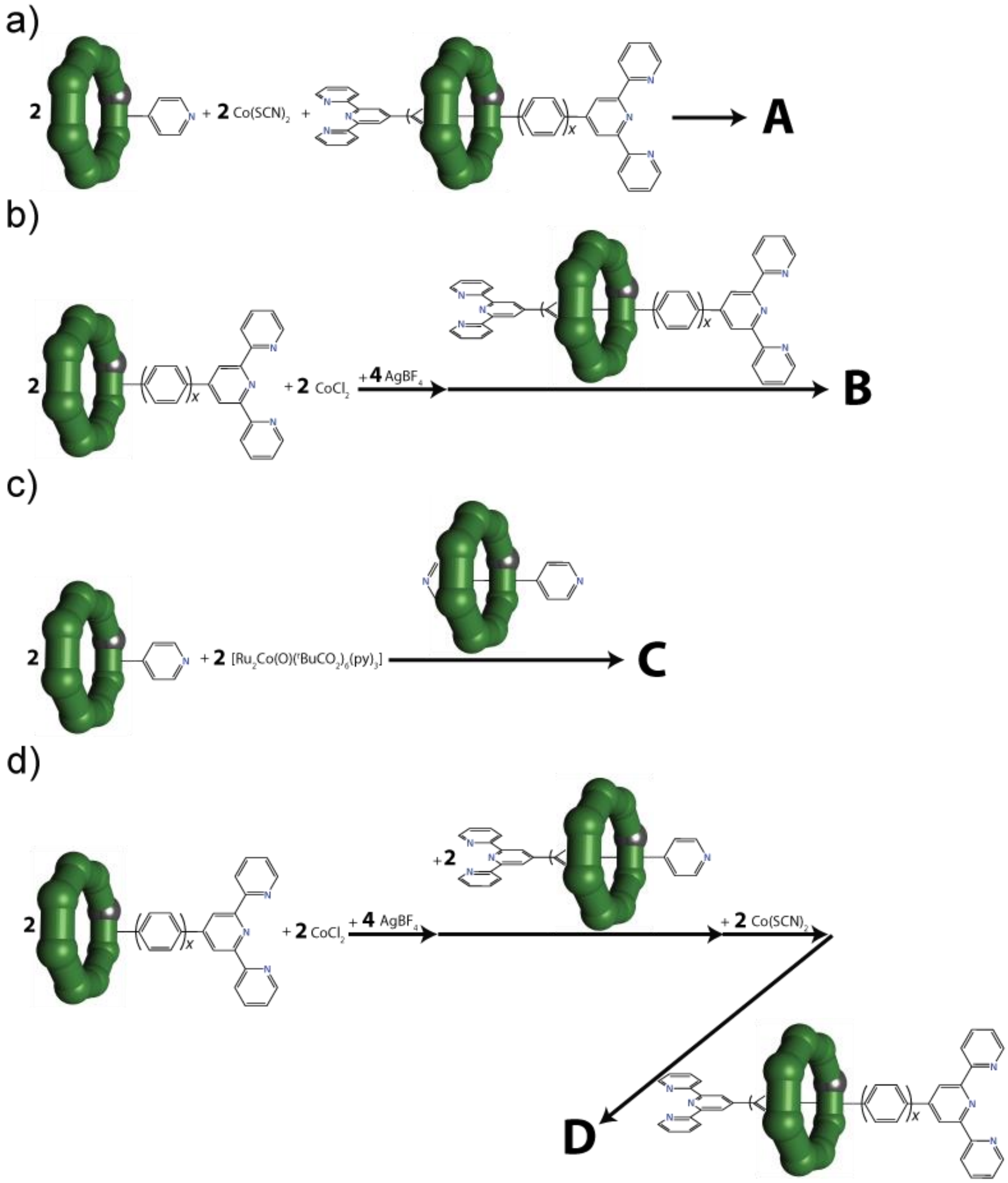

Supplementary Figure 31. Schematic representation of the experimental procedure to obtain the different finite chains of qubits.

Supplementary Tables

Supplementary Table 1. Crystallographic information for compounds **3**–**6**$^{\mathbf{ox}}$.

	**3**	**4**	**5**	**6**	**6**$^{\mathbf{ox}}$
Crystal colour	Green	Green	Green	Violet	Violet
Crystal size (mm)	0.7 × 0.15 × 0.14	0.1 × 0.1 × 0.05	0.35 × 0.2 × 0.05	0.15 × 0.15 × 0.07	0.3 × 0.3 × 0.1
Crystal system	Monoclinic	Monoclinic	Triclinic	Monoclinic	Triclinic
Space group, *Z*	$P2_1/c$, *4*	$P2_1/n$, *4*	P-1, *2*	$P2_1/n$, *8*	P-1, *2*
a (Å)	16.7978(4)	30.5037(19)	16.6948(3)	22.3181(5)	11.4660(5)
b (Å)	31.2594(11)	16.5057(5)	29.8156(6)	23.1069(4)	14.9252(9)
c (Å)	24.9128(9)	32.2556(19)	31.7036(6)	22.5654(6)	18.9394(8)
α (°)	90	90	70.4222(18)	90	84.303(4)
β (°)	103.209(3)	115.872(7)	83.8761(16)	112.263(3)	88.505(4)
γ (°)	90	90	77.9945(18)	90	68.403(5)
V (Å^3)	12735.3(7)	14612.5(15)	14531.7(5)	10769.6(4)	2998.5(3)
Density (Mg.m^{-3})	1.288	1.199	1.147	1.383	1.496
Wavelength (Å)	0.71073	1.54184	1.5418	0.71073	0.6889
Temperature (K)	150	100	100	150	100
μ(Mo-Kα) (mm^{-1})	0.795	4.880	5.442	0.916	0.890
2Θ range (°)	6.05 to 52.744	6.16 to 136.49	5.4162 to 149.1186	5.988 to 58.114	5.296 to 51.222
Reflns collected	44720	125577	238142	80327	14377
Independent reflns (R_{int})	25814 (0.0384)	26427 (0.1549)	55952 (0.1202)	25077 (0.0492)	14377 (0.176)
L.S. parameters, *p*	1554	2128	3030	1399	679
No. of restraints, *r*	445	2353	10938	382	159
$R1$ $(F)^a$ $I > 2.0\sigma(I)$	0.0569	0.1191	0.0697	0.0500	0.1192
$wR2(F^2)$,^{a} all data	0.1341	0.3876	0.1984	0.1295	0.3372
$S(F^2)$,^{a} all data	1.025	1.147	1.036	1.034	1.435

^{a} $R1(F) = \Sigma(|F_o| - |F_c|)/\Sigma|F_o|$; [b] $wR^2(F^2) = [\Sigma w(F_o^2 - F_c^2)^2/\Sigma wF_o^4]^{½}$; [c] $S(F^2) = [\Sigma w(F_o^2 - F_c^2)^2/(n + r - p)]^{½}$

Supplementary Table 2. Crystallographic information for compounds **7**–**11**.

	**7**	**8a**	**9a**	**10**	**11**
Crystal colour	Green	Green	Green	Red	Red
Crystal size (mm)	0.35 × 0.15 × 0.1	0.1 × 0.1 × 0.1	0.1 × 0.1 × 0.1	0.06 × 0.04 × 0.03	0.08 × 0.05 × 0.04
Crystal system	Hexagonal	Triclinic	Monoclinic	Monoclinic	Triclinic
Space group, *Z*	$P6_122$, *6*	*P-1, 2*	C2/c, *4*	C2/c, *8*	P-1, *2*
a (Å)	24.8796(5)	19.3835(3)	56.308(3)	40.103(2)	9.2332(6)
b (Å)	24.8796(5)	27.6538(4)	16.4647(3)	9.0797(4)	12.2853(9)
c (Å)	96.7732(16)	29.1765(6)	30.7417(9)	21.8936(12)	19.3386(11)
α (°)	90	76.8220(16)	90	90	85.098(5)
β (°)	90	84.5616(15)	93.238(3)	106.060(6)	76.757(5)
γ (°)	120	89.7001(13)	90	90	88.790(5)
V (Å^3)	51877(2)	15156.9(5)	28454.9(16)	7660.9(7)	2127.5(2)
Density (Mg.m^{-3})	1.074	1.205	1.249	1.499	1.529
Wavelength (Å)	1.54184	0.6889	0.6889	1.54178	1.54184
Temperature (K)	100	100	100	100	100
μ(Mo-Kα) (mm^{-1})	5.198	0.748	0.795	4.355	4.511
2Θ range (°)	4.202 to 136.496	2.928 to 49.038	3.192 to 49.038	4.586 to 136.464	4.71 to 117.868
Reflns collected	172691	126080	89423	27391	28635
Independent reflns (R_{int})	30634 (0.1702)	54261 (0.0586)	25476 (0.0874)	6898 (0.0817)	5992 (0.0930)

L.S. parameters, *p*	1463	3559	1898	595	652
No. of restraints, *r*	825	2007	5023	185	123
$R1$ $(F)^a$ $I > 2.0\sigma(I)$	0.1276	0.1050	0.1272	0.0831	0.1045
$wR2(F^2)$,[a] all data	0.3266	0.3574	0.4220	0.2723	0.3166
$S(F^2)$,[a] all data	1.308	1.199	1.301	1.020	1.040

[a] $R1(F) = \Sigma(|F_o| - |F_c|)/\Sigma|F_o|$; [b] $wR^2(F^2) = [\Sigma w(F_o^2 - F_c^2)^2/\Sigma wF_o^4]^{½}$; [c] $S(F^2) = [\Sigma w(F_o^2 - F_c^2)^2/(n + r - p)]^{½}$

Supplementary Table 3. Selected electrochemical[a] data for **6**, **7**, **8b**, **9b**, **10** and **11**.

Compound	E^b / V
**6**	–0.31(81)
**7**	–0.35(87)
**8b**	–0.30(86)
**9b**	–0.27(88)
**10**	–0.27(92)
**11**	–0.24(90)

[a] In dichloromethane (25 °C, 0.1 M nBu$_4$NPF$_6$) with a scan rate of 100 mV s^{-1}. [b] All formal potential (E) values were taken as the half-wave potentials *vs*. Ferrocene. The values of the peak-to-peak separation (ΔE / mV) between the anodic and cathodic peak potentials are given in parentheses.

Supplementary Table 4. Selected pulsed-EPR[a] data for **5**, **7**, **8b** and **9b**.

Compound	T / K	T_1 / ns	T_{SD} / ns	T_M / ns	s
**5**	2.6	17728 ± 325	4721 ± 322	683 ± 1	1.610 ± 0.002
**7**	2.7	24103 ± 1113	6987 ± 786	749 ± 1	1.696 ± 0.003
**7^{ox}**	2.5	23673 ± 91	–	767 ± 1	1.665 ± 0.003
**8b**	2.4	38668 ± 730	11367 ± 442	790 ± 1	1.658 ± 0.004
**8b^{ox}**	3.0			984 ± 1	1.283 ± 0.001
**9b**	2.8	15417 ± 440	–	750 ± 1	1.599 ± 0.005[b]
**9b^{ox}**	3.0			1031 ± 5	1.304 ± 0.008

[a] Measurements performed on a Bruker ElexSys E580 spectrometer operating at Q-band frequency (*ca.* 34 GHz), in diluted (0.0001 M) toluene solutions at the maximum resonance field for the ring in each compound. T_1 represents the spin-lattice relaxation time constant, T_{SD} the spectral diffusion time constant, T_M the phase memory time and s is a stretching parameter. [b] For B =13070 G we obtain T_M = 601 ± 3 and s = 1.169 ± 0.007.

Supplementary Table 5. Selected pulsed-EPR[a] data for **5**.

B / mT	*T* / K	T_M / ns	*s*
556 (◆)	2.4	470 ± 3	1
709 (◆)	2.4	499 ± 2	1
740 (◆)	2.4	487 ± 4	1
1247 (◆)	2.4	668 ± 1	1.591 ± 0.005
1350 (◆)	2.4	686 ± 2	1.670 ± 0.012

[a]Measurements performed on a Bruker ElexSys E580 spectrometer operating at Q-band frequency (*ca.* 34 GHz), in toluene solutions (0.002 M) at some representative resonance fields. T_M represents the phase memory time and *s* is a stretching parameter.

Supplementary Table 6. Selected pulsed-EPR[a] data for **7**.

B / mT	*T* / K	T_M / ns	*s*
557 (●)	2.6	666 ± 2	1
557 (●)	3.0	499 ± 2	1
557 (●)	3.3	439 ± 1	1
557 (●)	5.0	245 ± 5	1
776 (●)	2.6	706 ± 3	1
1260 (●)	2.6	693 ± 3	1.655 ± 0.014
1350 (●)	2.6	683 ± 5	1.568 ± 0.033

[a]Measurements performed on a Bruker ElexSys E580 spectrometer operating at Q-band frequency (*ca.* 34 GHz), in toluene solutions (0.002 M) at some representative resonance fields. T_M represents the phase memory time and *s* is a stretching parameter.

Supplementary Table 7. Selected pulsed-EPR[a] data for **8b**.

B / mT	*T* / K	T_M / ns	*s*
1166 (▲)	2.7	628 ± 4	1
1166 (▲)	5.0	541 ± 4	1
1281 (▲)	2.7	426 ± 1	1
1350 (▲)	2.7	692 ± 1	1.589 ± 0.003

[a]Measurements performed on a Bruker ElexSys E580 spectrometer operating at Q-band frequency (*ca.* 34 GHz), in toluene solutions (0.002 M) at some representative resonance fields. T_M represents the phase memory time and *s* is a stretching parameter.

Supplementary Table 8. Selected pulsed-EPR[a] data for **9b**.

B / mT	T / K	T_M / ns	s
1166 (■)	2.8	652 ± 4	1
1188 (■)	2.8	644 ± 2	1
1307 (■)	2.8	601 ± 3	1.169 ± 0.007
1366 (■)	2.8	709 ± 2	1.565 ± 0.010

[a]Measurements performed on a Bruker ElexSys E580 spectrometer operating at Q-band frequency (*ca.* 34 GHz), in toluene solutions (0.002 M) at some representative resonance fields. T_M represents the phase memory time and s is a stretching parameter.

Supplementary Table 9. Selected pulsed-EPR[a] data for **7^{ox}**, **8b**, **8b^{ox}** and **9b^{ox}**.

Compound	T / K	T_1 / ns	T_{SD} / ns	T_M / ns	s
**7^{ox}**	2.5	37533 ± 110	14799 ± 147	672 ± 1	1.481 ± 0.003[b]
**8b**	2.4	34044 ± 580	8180 ± 123	594 ± 2	1.402 ± 0.007
**8b^{ox}**	2.4	47331 ± 87	13970 ± 61	584 ± 1	1.391 ± 0.003
**9b^{ox}**	2.5	37585 ± 78	9473 ± 58	450 ± 1	1.239 ± 0.002

[a] Measurements performed on a Bruker ElexSys E580 spectrometer operating at X-band frequency (*ca.* 9.5 GHz), in diluted (0.0001 M) toluene solutions at the maximum resonance field for the ring in each compound. T_1 represents the spin-lattice relaxation time constant, T_{SD} the spectral diffusion time constant, T_M the phase memory time and s is a stretching parameter. [b] For π = 64 ns and τ = 180 ns, T_M = 758 ± 1 and s = 1.581 ± 0.002.

Supplementary Table 10. Selected pulsed-EPR[a] data for **7**.

B / mT	T / K	T_M / ns	s
161 (●)	2.7	482 ± 2	1
214 (●)	2.7	528 ± 1	1
244 (●)	2.7	571 ± 1	1
365 (●)	2.7	613 ± 1	1.469 ± 0.002
420 (●)	2.7	635 ± 1	1.532 ± 0.003

[a]Measurements performed on a Bruker ElexSys E580 spectrometer operating at X-band frequency (*ca.* 9.5 GHz), in toluene solutions (0.002 M) at some representative resonance fields. T_M represents the phase memory time and s is a stretching parameter.

Supplementary Table 11. Selected pulsed-EPR[a] data for **8b**.

B / mT	*T* / K	T_M / ns	*s*
322 (▲)	2.7	608 ± 7	1
338 (▲)	2.7	575 ± 7	1
390 (▲)	2.7	596 ± 1	1.620 ± 0.004

[a]Measurements performed on a Bruker ElexSys E580 spectrometer operating at X-band frequency (*ca.* 9.5 GHz), in toluene solutions (0.002 M) at some representative resonance fields. T_M represents the phase memory time and *s* is a stretching parameter.

Supplementary Table 12. Selected pulsed-EPR[a] data for **9b**.

B / mT	*T* / K	T_M / ns	*s*
318 (■)	2.6	388 ± 7	1
341 (■)	2.6	379 ± 4	1
370 (■)	2.6	640 ± 9	1.695 ± 0.054
390 (■)	2.6	618 ± 1	1.496 ± 0.002

[a]Measurements performed on a Bruker ElexSys E580 spectrometer operating at X-band frequency (*ca.* 9.5 GHz), in toluene solutions (0.002 M) at some representative resonance fields. T_M represents the phase memory time and *s* is a stretching parameter.

Supplementary Methods

Synthetic details

Unless stated otherwise, all reagents and solvents were purchased from Aldrich Chemicals and used without further purification. [n-Pr_2NH_2][$Cr_7Ni(\mu\text{-}F)_8(O_2C^tBu)_{16}$] (**1**), was prepared according to literature procedure,[1] but with [$2NiCO_3\cdot3Ni(OH)_2\cdot4H_2O$] as the source of Ni(II) instead of the compound [$Ni_2(\mu\text{-}OH_2)(O_2C^tBu)_4(HO_2C^tBu)_4$] used in the original method, and with a reaction time of 24 h at 160 ºC. Functionalised ring with *iso*-nicotinic acid [$^nPr_2NH_2$][$Cr_7NiF_8(O_2C^tBu)_{15}(O_2C\text{-py})$] **2** and 4-Furane-2,2':6',2"-terpyridine, were obtained following reported procedure.[2,3] Column chromatography was carried out using Silica 60 A (particle size 35-70 μm, Fisher, UK) as the stationary phase, and TLC was performed on pre-coated silica gel plates (0.25 mm thick, 60 F_{254}, Merck, Germany) and observed under UV light. NMR spectra were recorded on Bruker AV 400 instrument. Chemical shifts are reported in parts per million (ppm) from low to high frequency and referenced to the residual solvent resonance. ESI mass spectrometry and microanalysis were carried out by the services at the University of Manchester.

Synthesis of ligands

4-carboxy-2,2':6',2"-terpyridine (HO_2C-terpy)

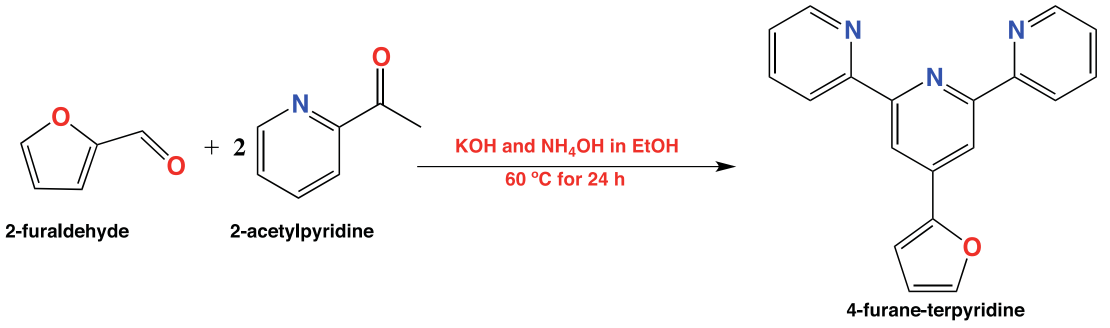

4-Furane-2,2':6',2"-terpyridine: 2-furaldehyde (1.92 g, 20 mmol) and 2-acetyl-pyridine (4.84 g, 40 mmol) were dissolved in EtOH (100 mL). To this was added KOH (3.08 g, 55 mmol) and aqueous ammonia solution (50 mL, 25%). The solution was then stirred at 60 °C for 24 h after which about half the solvent was removed under reduced pressure yielding an off-white product (5.30 g, 88%). ^{1}H-NMR (400 MHz, 298K, $CDCl_3$): δ = 6.57 (dd, 1H), 7.11 (d, 1H), 7.35 (ddd, 2H), 7.59 (d, 1H), 7.87 (dt, 2H), 8.64 (d, 2H), 8.72 (s, 2H), 8.74 (d, 2H). ^{13}C-NMR (75 MHz, 298K, $CDCl_3$): δ = 108.6; 110.7; 114.2; 121.0; 123.4; 136.1; 138.8; 142.5; 149.50; 151.2; 153.3; 157.6. ESI-MS (m/z): +300 $[M + H]^+$ (100%); +32 $[M + Na]^+$ (100%); −298 $[M - H]^-$ (10%). Elemental analysis (% calcd., % found for $C_{19}H_{13}N_3O$): C (76.24, 76.44), H (4.38, 4.38), N (14.04, 13.94).

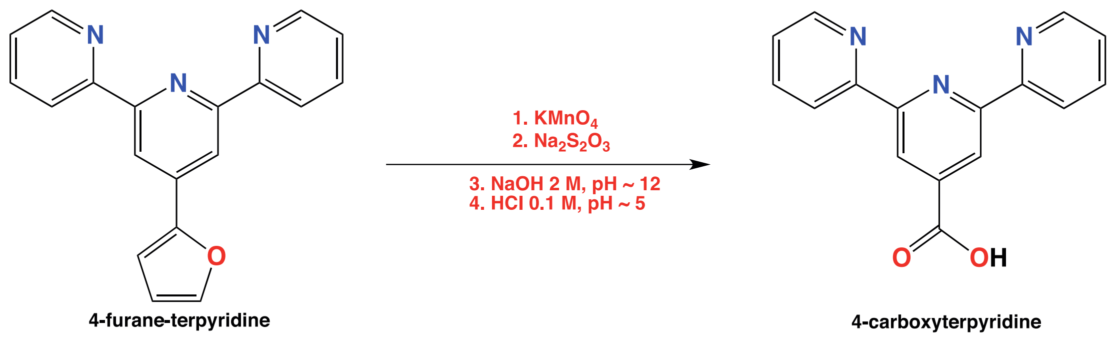

4-carboxy-2,2':6',2"-terpyridine: to a solution of 4-furane-terpyridine (2.20 g, 7.3 mmol) in pyridine (40 mL) and H_2O (30 mL) was added $KMnO_4$ (7.89 g, 50 mmol) in stepwise portions and then stirred for 72 h. Residual $KMnO_4$ was reduced by adding $Na_2S_2O_3$ in H_2O until the violet colour of the mixture disappeared. The solution was then made basic by the addition of NaOH (2 M) and the resultant MnO_2 formed was filtered off. The solvent was removed from the filtrate *in vacuo* and then reconstituted in NaOH (pH ≈ 12). The resultant suspension was acidified with HCl (0.1 M) filtered and the white solid was collected (1.86 g, 92%). ^{1}H-NMR (400 MHz, 298K, DMSO-d_6): δ 7.51–7.55 (m, 2H), 8.03 (dt, 2H), 8.64 (dd, 2H), 8.74–8.76 (m, 2H), 8.85 (s, 2H). ^{13}C-NMR (75 MHz, 298K, CD_3OD): δ = 121.3; 122.4; 125.1; 138.6; 148.5; 150.1; 156.9; 156.7; 172.0. ESI-MS (*m*/*z*): +300 $[M + Na]^+$ (100%); +278 $[M + H]^+$ (50%); −276 $[M - H]^-$ (100%). Elemental analysis (% calcd., % found for $C_{16}H_{11}N_3O_2$): C (69.31, 69.28), H (3.99, 4.02), N (15.15, 15.12).

4′-(4-carboxyphenyl)-2,2′:6′,2″-terpyridine (HO_2C-Ph-terpy)

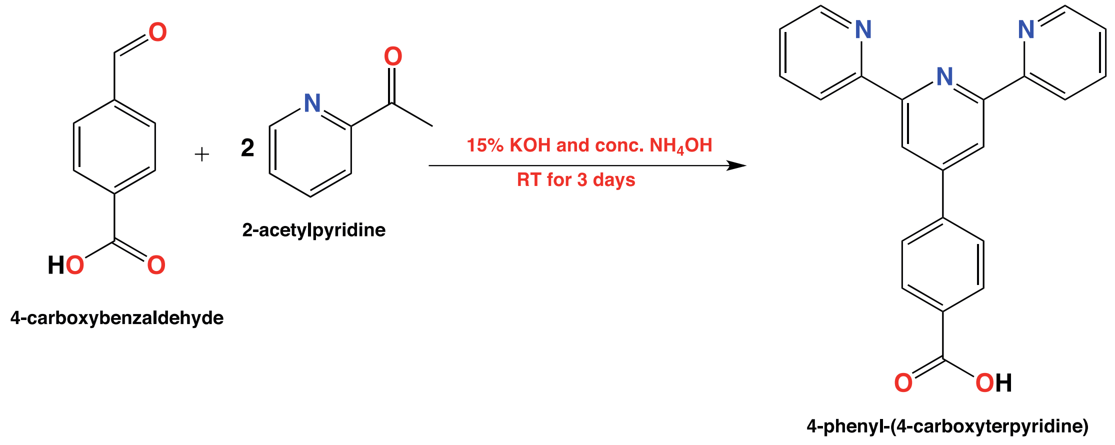

4-phenyl-(4-carboxyterpyridine): 2-acetyl-pyridine (2.90 g, 24 mmol) and 4-carboxybenzaldehyde (1.80 g, 12 mmol) were dissolved in MeOH (120 mL) by stirring for 30 min, followed by the addition of *15% KOH (80 mL) and concentrated NH_4OH (*15 *mL). The mixture was allowed to stand at ambient temperature for 3 d. The emulsion that formed was filtered off and washed with $CHCl_3$ (*60 *mL) and cold MeOH/H_2O (1:1,* 60 *mL). The crude product was suspended in MeOH/H_2O (80:20), and the mixture was stirred sonicated at* 40 *ºC until a clear solution was obtained. This was then acidified to pH = 2 by addition of HCl (1 M), resulting in the formation of a white precipitated that was collected by vacuum filtration and rinsed with cold water and then dried (6.70 g, 79%). ^{1}H-NMR (400 MHz,* 298K, *[D]tfa/D_2O): δ 7.90 (d, 2H), 8.13 (dd, 2H), 8.30 (d, 2H), 8.83 (d, 2H), 8.65 (dd, 2H), 8,74 (s, 2H), 9.04 (d, 2 H).* ^{13}C-NMR (75 MHz, 298K, [D]tfa/D_2O): δ = 125.4; 126.9; 129.7; 132.3; 133.0; 133.3; 142.6; 143.8; 149.0; 149.4; 150.1; 156.2; 174.1. *ESI-MS (m/z): +353 $[M + H]^+$ (100%). Elemental analysis (% calcd., % found for $C_{22}H_{15}N_3O_2$): C (74.78, 74.63), H (4.28, 4.33), N (11.89, 11.84).*

Synthesis of functionalised {Cr_7Ni} ring

$[^nPr_2NH_2][Cr_7NiF_8(O_2C^tBu)_{15}(O_2C\text{-terpy})]$ **3**

An excess of 4-carboxyterpyridine (1.30 g, 4 mmol) was reacted with **1** (3 g, 1.3 mmol) in toluene (25 mL) and 1,2-dichlorobenzene (75 mL). The solution was heated with constant stirring at 160 ºC for 4 h in a round-bottomed flask. Solvent was removed under reduced pressure and the resulting residue was purified by column chromatography. First toluene, followed by 60:1 toluene:ethyl acetate was used, which allowed un-reacted $[^nPr_2NH_2][Cr_7Ni(\mu\text{-}F)_8(O_2C^tBu)_{16}]$ to be eluted, leaving the products of the reaction at the top of the column. Thereafter 10:1 toluene:ethyl acetate was used, eluting **3**. The solvent was removed under reduced pressure and X-ray quality crystals were obtained from recrystallization from Et_2O/MeCN (1.20 g, 37 %). ESI-MS (*m*/*z*): +2493 $[M + Na]^+$ (100%); +1258 $[M + 2Na]^{2+}$ (60%); −2368 $[M - {}^nPr_2NH_2]^-$ (100%); −2368 $[M - {}^nPr_2NH_2]^-$ (100%). Elemental analysis (% calcd., % found for $C_{97}H_{161}Cr_7F_8N_4NiO_{32}$): C (47.17, 47.25), H (6.57, 6.58), Cr (14.17, 14.23), N (2.27, 2.30), Ni (2.38, 2.27).

$[^nPr_2NH_2][Cr_7NiF_8(O_2C^tBu)_{15}(O_2C\text{-Ph-terpy})]$ **4**

An excess of 4-phenyl-(4-carboxyterpyridine) (1.40 g, 4 mmol) was reacted with **1** (3 g, 1.3 mmol) in toluene (25 mL), DMF (20 mL) and 1,2-dichlorobenzene (75 mL). The solution was heated with constant stirring at 160 ºC for 5 h in a round-bottomed flask. Solvent was removed under reduced pressure and the resulting residue was purified by column chromatography. First toluene, followed by 60:1 toluene:ethyl acetate was used, which allowed un-reacted $[^nPr_2NH_2][Cr_7Ni(\mu\text{-}F)_8(O_2C^tBu)_{16}]$ to be eluted, leaving the products of the reaction at the top of the column. Thereafter 10:1 toluene:ethyl acetate was used, eluting **4**. The solvent was removed under reduced pressure and X-ray quality crystals were obtained from vapour diffusion of MeCN into a Toluene solution of **4** (0.72 g, 21%). ESI-MS (*m*/*z*): +2467 $[M + Na - NH_2n\text{-}Pr_2]^+$ (40%); +1258 $[M + 2Na - NH_2n\text{-}Pr_2]^{+2}$ (70%); −2368 $[M - 2NH_2n\text{-}Pr_2 + Na]^-$ (100%). Elemental analysis (% calcd., % found for $C_{110}H_{173}Cr_7F_8N_4NiO_{32}$): C (49.69, 50.08), H (6.58, 6.61), Cr (13.65, 13.80), N (2.08, 2.12), Ni (2.25, 2.23).

Synthesis of $[Ru^{III}_2Co^{II}O(^tBuCO_2)_6(py)_3]^n$ (n = 0 and +1) triangles

$[Ru^{III}_2Co^{II}O(^tBuCO_2)_6(py)_3]$ **6**

A mixture of $RuCl_3 \cdot xH_2O$ (0.15 g; 0.57 mmol) and pivalic acid (22.1 g; 219 mmol) in 4:1 EtOH/H_2O (25 mL) was heated for 10 min at 70 °C. After cooling to room temperature, $[Co_2(H_2O)(^tBuCO_2)_4(^tBuCO_2H)_4]$ (0.95 g; 2.01 mmol) was added, and the reaction mixture stirred until all had dissolved and the colour changed from red to purple. The solution was left to stand overnight and then filtered. The solvent was removed by rotary evaporation and the resultant oil was filtered to remove excess metal pivalate, treated with MeCN (10 mL) then placed in the freezer (-40 °C) overnight. Repeated freeze-filter cycles ensured the removal of excess pivalic acid. The solvent was then stripped under vacuum before distilled water (200 mL) was added to the purple residue, and stirred for 30 min. The solution was filtered to yield a dark purple solid, which was washed with 4:1 H_2O/MeCN (3 × 10 mL) and dried in air. The solid was dissolved in MeCN (30 mL) and pyridine (10 mL), stirred overnight and filtered. The solvent was removed by rotary evaporation and the purple residue recrystallized from a minimum amount of pyridine (0.06 g, 19%). ^{1}H-NMR (400 MHz, $CDCl_3$): δ -6.73 (s, 18H), 7.52 (m, 2H), 7.95 (m, 4H), 9.30 (m, 4H), 16.77 (s, 36H). ESI-MS (*m*/*z*): +1066 $[M + Na - py]^+$. Elemental analysis (% calcd., % found for $C_{45}H_{69}O_{13}N_3CoRu_2$): C (48.21, 48.69), H (6.20, 6.34), N (3.75, 3.72), Co (5.26, 5.21).

$[Ru^{III,IV}_2Co^{II}O(^tBuCO_2)_6(py)_3](PF_6)$ $\mathbf{6^{ox}}$

Under an N_2 atmosphere, a dark purple solution of **6** (100 mg; 0.09 mmol) in CH_2Cl_2 (20 mL) was combined with $[FeCp_2](PF_6)$ (0.03 g; 0.08 mmol) and stirred for 30 min. The deep purple solution was left to stand for 10 min before being filtered. Hexane (20 mL) was added, the solution concentrated to a third of the volume and placed in the freezer (-40 °C) to crystallize. (0.06 g, 56%). ^{1}H-NMR (400 MHz, $CDCl_3$): δ -0.61 (s, 18H), 3.89 (s, 36H), 6.54 (m, 4H), 7.62 (m, 4H), 9.60 (m, 2H). ESI-MS (*m*/*z*): +1043 $[M - py]^+$. Elemental analysis (% calcd., % found for $C_{45}H_{69}O_{13}N_3F_6PCoRu_2$): C (42.69, 42.40), H (5.49, 5.38), N (3.32, 3.19), P (2.45, 2.28), Co (4.69, 4.02).

Synthesis of two-qubits supramolecular assemblies

$\{[^nPr_2NH_2][Cr_7NiF_8(O_2C^tBu)_{15}(O_2C$-py$)]Co(NCS)_2[^nPr_2NH_2][Cr_7NiF_8(O_2C^tBu)_{15}(O_2C$-terpy$)]\}\cdot 2.5Me_2CO$ **5**

To a solution of **2** (0.100 g, 0.04 mmol) and **3** (0.099 g, 0.04 mmol) in diethyl ether/acetone (1/1; 15 mL) was added dropwise a solution of $Co(SCN)_2$ (7 mg, 0.04mmol) in hot acetone (5 mL) and the solution was stirred for 1 h at 60 ºC. The brownish-green solution was then filtered and kept at room temperature undisturbed until dark-green crystals suitable for X-ray diffraction appeared (0.172 g, 85%). Elemental analysis (% calcd., % found for $C_{192.5}H_{329.5}CoCr_{14}F_{16}N_8Ni_2O_{66}S_2$): C (45.47, 45.49), H (6.53, 6.58), S (1.26, 1.30), Co (1.16, 1.19), Cr (14.32, 14.34), N (2.20, 2.23), Ni (2.31, 2.34).

$[\{[^{n}Pr_2NH_2][Cr_7NiF_8(O_2C^{t}Bu)_{15}(O_2C\text{-}py)]\}_2[Ru^{III}{}_2Co^{II}O(^{t}BuCO_2)_6(py)]]$ **7**

To a solution of **2** (0.100 g, 0.04 mmol) in acetone (15 mL) was added dropwise a solution of **6** (0.022 g, 0.02 mmol) and the solution was stirred for 6 h at 60 ºC. The purple solution was then filtered and kept at room temperature undisturbed until purple crystals suitable for X-ray diffraction appeared (0.097 g, 87%). Elemental analysis (% calcd., % found for $C_{269}H_{369}CoRu_2Cr_{14}F_{16}N_5Ni_2O_{77}$): C (44.87, 45.09), H (6.65, 6.68), Co (1.05, 1.09), Cr (13.01, 13.04), N (1.25, 1.23), Ni (2.10, 2.14).

$[\{[^{n}Pr_2NH_2][Cr_7NiF_8(O_2C^{t}Bu)_{15}(O_2C\text{-}terpy)]\}_2Co](ClO_4)_2$ **8a**

To a solution of **3** (0.395 g, 0.16 mmol) in diethyl ether/acetone (3/1; 15 mL) was added a solution of $Co(ClO_4)_2\cdot\square H_2O$ (29 mg, 0.08 mmol) in acetone (5 mL). The resulting solution was then stirred for 1 h at room temperature. The dark-brown solution was then filtered and the solvent was removed under reduced pressure. Dark-brown X-ray quality crystals were obtained from recrystallization from THF/MeCN (0.352 g, 80%). Elemental analysis (% calcd., % found for $C_{216}H_{347}Cl_2CoCr_{14}F_{16}N_9Ni_2O_{72}$): C (47.16, 47.30), H (6.36, 6.38), Cl (1.29, 1.32), Co (1.07, 1.05), Cr (13.23, 13.34), N (2.29, 2.23), Ni (2.13, 2.20).

$[\{[^{n}Pr_2NH_2][Cr_7NiF_8(O_2C^{t}Bu)_{15}(O_2C\text{-}terpy)]\}_2Co](BF_4)_2$ **8b**

The compound **8b** was prepared by an analogous procedure to that for **8a** by using $Co(BF_4)_2\cdot\square H_2O$ as metal salt instead of $Co(ClO_4)_2\cdot\square H_2O$. Unfortunately, we were unable to get suitable crystals of **8b** for single crystal X-ray diffraction experiment (0.350 g, 84%). Elemental analysis (% calcd., % found for $C_{194}H_{322}B_2CoCr_{14}F_{24}N_8Ni_2O_{64}$): C (45.05, 44.89), H (6.28, 6.48), B (0.42, 0.38), Co (1.14, 1.15), Cr (14.07, 13.74), N (2.17, 2.19), Ni (2.27, 2.32).

$[\{[^{n}Pr_2NH_2][Cr_7NiF_8(O_2C^{t}Bu)_{15}(O_2C\text{-}Ph\text{-}terpy)]\}_2Co](ClO_4)_2$ **9a**

To a solution of **4** (0.422 g, 0.16 mmol) in diethyl ether/acetone (3/1; 15 mL) was added a solution of $Co(ClO_4)_2\cdot\square H_2O$ (29 mg, 0.08 mmol) in acetone (5 mL). The resulting solution was then stirred for 1 h at room temperature. The dark-brown solution was then filtered and the solvent was removed under reduced pressure. Dark-brown X-ray quality crystals were obtained from recrystallization from THF/MeCN (0.321 g, 75%). Elemental analysis (% calcd., % found for $C_{206}H_{330}Cl_2CoCr_{14}F_{16}N_8Ni_2O_{72}$): C (46.16, 46.25), H (6.26, 6.22), Cl (1.30, 1.32), Co (1.12, 1.10), Cr (13.63, 13.61), N (2.06, 2.09), Ni (2.15, 2.19).

$[\{[^{n}Pr_2NH_2][Cr_7NiF_8(O_2C^{t}Bu)_{15}(O_2C\text{-}Ph\text{-}terpy)]\}_2Co](BF_4)_2$ **9b**

The compound **9b** was prepared by an analogous procedure to that for **9a** by using $Co(BF_4)_2\cdot\square H_2O$ as metal salt instead of $Co(ClO_4)_2\cdot\square H_2O$. Unfortunately, we were unable to get suitable crystals of **9b** for single crystal X-ray diffraction experiment (0.350 g, 82%). Elemental analysis (% calcd., % found for $C_{206}H_{330}B_2CoCr_{14}F_{24}N_8Ni_2O_{64}$): C (46.25, 46.47), H (6.23, 6.25), B (0.40, 0.41), Co (1.13, 1.11), Cr (13.77, 13.67), N (2.11, 2.10), Ni (2.23, 2.21).

Synthesis of monometallic cobalt complexes

[Co(HO₂C-terpy)₂](BF₄)₂ **10**

To a solution of 4-carboxyterpyridine (0.100 g, 0.36 mmol) in MeOH/DCM (1/1; 5 mL) was added $Co(BF_4)_2$·□H_2O (61 mg, 0.18 mmol). The solution was then stirred for 1 h. The dark-red solution was then filtered and kept at room temperature undisturbed. X-ray quality crystals were obtained after 24 h from slow evaporation of the solvent (0.122 g, 79%). ESI-MS (*m*/*z*): +613 $[M-2BF_4+H]^+$ (40%); +307 $[M-2BF_4]^{2+}$ (100%). Elemental analysis (% calcd., % found for $C_{34}H_{27}B_2CoF_8N_6O_7$): C (47.46, 47.25), H (3.23, 3.15), B (2.48, 2.50), Co (6.67, 6.82), N (9.59, 9.73).

[Co(HO₂C-Ph-terpy)₂](BF₄)₂ **11**

To a solution of 4-phenyl-(4-carboxyterpyridine) (99 mg, 0.28 mmol) in a solution of MeOH/DCM (1:1) was added $Co(BF_4)\cdot H_2O$ (48 mg, 0.14 mmol). The solution was then stirred for 1 h. The dark-red solution was then filtered and kept at room temperature undisturbed. X-ray quality crystals were obtained after 24 h from slow evaporation of the solvent (0.112 g, 82%). ESI-MS (*m*/*z*): +767 $[M-2BF_4+H]^+$ (20%); +383 $[M-2BF_4]^{+2}$ (100%). Elemental analysis (% calcd., % found for $C_{44.5}H_{29}B_2ClCoF_8N_6O_4$): C (54.31, 54.27), H (3.04, 3.00), B (2.25, 2.22), Co (6.18, 6.05), N (8.76, 8.63).

Crystallography

Data Collection. X-ray data was collected at a temperature of 150 K on an Agilent Technologies Supernova diffractometer with $Mo_{K\alpha}$ radiation, (λ = 0.71073 Å) for compounds **3** and **6,** equipped with an Oxford Cryosystems Cobra nitrogen flow gas system. Data was measured using CrysAlisPro suite of programs. X-ray data for compounds **4, 5, 7, 10** and **11** was collected at a temperature of 100 K using a Bruker X8 Prospector diffractometer with Cu-Kα radiation (λ = 1.54184 Å), equipped with an Oxford Cryosystems Cobra nitrogen flow gas system. Data was measured using Bruker APEX2 suite of programs. Synchrotron X-ray data were collected at beamline I19 (λ = 0.6889 Å) Diamond Light Source,[4] for compounds **6^{ox}**, **8a** and **9a** at temperature of 100 K. Data were measured using CrystalClear-SM Expert 2.0 r5 suite of programs.

Crystal structure determinations and refinements. X-ray data for compounds **3-11** were processed and reduced using CrysAlisPro suite of programs. Absorption correction was performed using empirical methods based upon symmetry-equivalent reflections combined with measurements at different azimuthal angles.[5,6] The crystal structure was solved and refined against all F^2 values using the SHELXTL suite of programs.[7,8]
Pivalate ligands were disorder in all the crystal structures. The C-C distances have been restrained using SADI, DFIX and SAME commands. The ADPs were also restrained using SIMU, RIGU and in some cases EADP commands. Hydrogen atoms were placed in calculated positions refined using idealized geometries (riding model)

and assigned fixed isotropic displacement parameters. The phenyl groups were restrained to have idealized geometries using AFIX commands.

Compounds **5**, **7** and **8a** present large voids filled with a lot of scattered electron density, the SQUEEZE protocol inside PLATON suites was used to account the void electron density.[9]

CCDC 1029608-1029613 and 1415380-1415383 contain the supplementary crystallographic data for this paper. These data can be obtained free of charge *via* www.ccdc.cam.ac.uk/conts/retrieving.html (or from the Cambridge Crystallographic Data Centre, 12 Union Road, Cambridge CB21EZ, UK; fax: (+44)1223-336-033; or deposit@ccdc.cam.ac.uk).

Electrochemistry

Cyclic voltammograms were recorded using 0.1 M $n\mathrm{NBu_4PF_6}$ as supporting electrolyte and 1.0 mM of **6**, **7**, **8b**, **9b**, **10** and **11** in dried DCM (Supplementary Figure 4 and Table S3). The working electrode was a glassy carbon disk (0.32 cm^2) that was polished with 1.0 µm diamond powder, sonicated, washed with absolute ethanol and acetone, and air dried. The reference electrode was $AgClO_4/Ag$ separated from the test solution by a salt bridge containing the solvent/supporting electrolyte, with platinum as auxiliary electrode. All experiments were performed in standard electrochemical cells at 25 °C under nitrogen. The investigated potential range was in the range of –2.00 to +1.80 V *vs*. SCE. The formal potentials were measured at a scan rate of 100 mV s^{-1} and they were referred to the internal standard ferrocene/ferrocenium couple [$E(Fc^+/Fc)$ = +0.46 V *vs*. SCE, ($\Delta E_p(Fc^+/Fc)$ = 80 mV; CH_2Cl_2, 0.1 M $n\mathrm{Bu_4NPF_6}$, 25 °C)].[10]

Magnetic measurements

Variable-temperature (2.0–300K) magnetic susceptibility measurements under an applied field of 1000 Oe and variable-field (0–7.0 T) magnetization measurements were carried out on powdered samples of **5**, **7**, **8b** and **9b** either constrained in eicosane with a Quantum Design MPMS-XL7 SQUID magnetometer (Supplementary Figures 5–9). The experimental data were corrected for the diamagnetism of the compounds (Pascal constants) and for diamagnetic contribution of eicosane and the sample holder.

Electron Paramagnetic Resonance

X-band (*ca.* 9.5 GHz), K-band (*ca.* 24 GHz), Q-band (*ca.* 34 GHz) and W-band (*ca.* 94 GHz) EPR spectra of **5** were recorded with a Bruker EMX580 spectrometer, Elexys 600 and EMX500 spectrometers. K-band (*ca.* 24 GHz) EPR spectra for **7**, Q-band (*ca.* 34 GHz) EPR spectra for **8b** and **9b** and X-band (*ca.* 9.5 GHz) EPR spectra for **10** and **11**, were recorded with a Bruker EMX580 spectrometer (Figure 3 and Supplementary Figures 10–13. The data was collected at 5 K using liquid helium. Spectral simulations were performed using the *EasySpin 4.5.5* simulation software.[11]

Pulsed electron paramagnetic resonance measurements were performed at low-temperatures on a Bruker ElexSys E580 spectrometer operating at X- (*ca.* 9.5 GHz) and Q-band frequency (*ca.* 34 GHz). 0.0001 M and/or 0.002 M toluene solutions of compounds were used (Supplementary Figures 14–27 and Supplementary Tables 4–13).

Inversion recovery

The inversion recovery pulse sequence used was π-t-π/2-τ-π-τ-echo, with π=32 ns, τ = 320 ns and variable *t*. The spin-lattice relaxation time constant, T_1, was deducted by fitting the resulting signal to Eq. 1:

$$I(t) = I_1 \exp(-t/T_1) + I_{SD} \exp(-t/T_{SD}) \qquad (1)$$

where I_1 and I_{SD} are the amplitudes and T_{SD} is the spectral diffusion time constant.

Phase memory time, T_M

The spin-echo decay measurements were carried out by gradually increasing the inter-pulse delay τ of a primary Hahn echo sequence π/2-τ-π-τ-*echo.* With microwave pulses of length π = 32 ns, strong proton-electron spin modulation was observed. In order to suppress such modulation, microwave pulses of length π = 128 ns were used. The phase memory time T_M could be deducted by fitting the experimental data to equation 2.

$$I(2\tau)=I(0)\exp[(-2\tau/T_M)^s] \qquad (2)$$

where *s* is a stretching parameter.[26]

DEER measurements

Double Electron-Electron Resonance (DEER) was measured for compounds $\mathbf{7^{ox}}$ and $\mathbf{9b^{ox}}$. All DEER experiments used a 4-pulse sequence with ν_1 detection pulses (π/2 and π) of 32 ns and ν_2 pump pulses of length 16 ns. Data were processed using DeerAnalysis.[12] Using this package, the raw data were smoothed, filtered to remove the effects of electron spin echo envelope modulation (ESEEM) arising from protons, and background-corrected to account for inter-dimer interactions in the three-dimensional homogenous distribution. The sample concentrations were sufficiently small that the background correction was at all times small.

Computational details

Fidelity of the simulated gates

We recall that the fidelity of a gate measures its performance, i.e., the degree to which the actual state evolution matches the ideal one. It is defined by $F = |\langle\psi_0|\psi(t_f)\rangle|$, where for a given starting logical state, $|\psi_0 >$ is the final state after an ideal gate, whereas $|\psi(t_f) >$ is the actual final state.

Quantum simulation of an antisymmetric Hamiltonian

Compound **5** is well suited for the quantum simulation of antisymmetric Hamiltonians. As an illustrative example, we apply a sequence of single and two-qubit quantum gates to simulate the evolution induced on two $S = {}^{1}/_{2}$ spins by the anti-symmetric (Dzyaloshinskii-Moriya) exchange interaction $H_a = G(S_1^z S_2^x - S_1^x S_2^z)$. The sequence of quantum gates required to implement the evolution induced by a Hamiltonian term of type $GS_1^z S_2^x$ is $R_y(\pi/2)e^{-iGtS_1^z S_2^z}R_y(-\pi/2)$, where $R_y(\theta)$ is a rotation of the second qubit about the Y-axis and the Ising $GS_1^z S_2^z$ evolution is obtained similarly to the C_ϕ gate, by simultaneously exciting and de-exciting both components |00> and |11> outside the computational basis to the corresponding states where the Co spin has been tilted. Supplementary Figure 28 shows that the calculated fidelity for the simulation of this model is high, and indeed the agreement with the exact evolution is evident. Here in order to monitor the time evolution induced by H_a on the two-qubit state we have reported the associated evolution of the average total spin of the two qubits $< S_1^z + S_2^z >$. In fact, this observable can be extracted in an actual experiment on an ensemble of molecules by a measurement of the sample magnetization.

We have also simulated the evolution due to the other terms of Dzyaloshinskii-Moriya interaction. In particular, for a term $GS_1^y S_2^x$ the sequence of gates to be implemented is $R_x^1(-\pi/2)R_y^2(\pi/2)e^{-iGtS_1^z S_2^z}R_y^2(-\pi/2)R_x^1(\pi/2)$. The obtained fidelity is of the order of 99.5% on a random superposition initial state.

Residual qubit-qubit coupling in compound 5

We determine the form of the unwanted residual effective ring-ring interaction in the low-energy subspace in which Co is in its *M* = -1/2 state (switch in the off state). By using second-order perturbation theory the Co-qubit coupling leads to an effective qubit-qubit interaction given by equation 3

$$H_{eff} = \Gamma_{xx} S_{1x} S_{2x} + \Gamma_{yy} S_{1y} S_{2y} + \lambda_1 S_{1z} + \lambda_2 S_{2z} + C \qquad (3)$$

$$\Gamma_{xx} = -\frac{g_{1z} J_{1y} J_{2x}\left[(g_{Coz})^2 - (g_{2z})^2\right] + J_{1x}\left\{J_{2x} g_{Coz}\left[2(g_{Coz})^2 - (g_{2z})^2 - (g_{1z})^2\right] + g_{2z} J_{2y}\left[(g_{Coz})^2 - (g_{1z})^2\right]\right\}}{\mu_B B_z\left[(g_{Coz})^2 - (g_{1z})^2\right]\left[(g_{Coz})^2 - (g_{2z})^2\right]}$$

$$\Gamma_{yy} = -\frac{g_{2z} J_{1y} J_{2x}\left[(g_{Coz})^2 - (g_{1z})^2\right] + J_{2y}\left\{J_{1y} g_{Coz}\left[2(g_{Coz})^2 - (g_{2z})^2 - (g_{1z})^2\right] + g_{1z} J_{1x}\left[(g_{Coz})^2 - (g_{2z})^2\right]\right\}}{\mu_B B_z\left[(g_{Coz})^2 - (g_{1z})^2\right]\left[(g_{Coz})^2 - (g_{2z})^2\right]}$$

$$\lambda_1 = J_{1z} - \frac{2g_{Coz} J_{1x} J_{1y} + g_{1z}\left[(J_{1x})^2 + (J_{1y})^2\right]}{2\mu_B B_z\left[(g_{Coz})^2 - (g_{1z})^2\right]}$$

$$\lambda_2 = J_{2z} - \frac{2g_{Coz} J_{2x} J_{2y} + g_{2z}\left[(J_{2x})^2 + (J_{2y})^2\right]}{2\mu_B B_z\left[(g_{Coz})^2 - (g_{2z})^2\right]}$$

Apart from a constant term and a renormalization of the external field felt by the qubits, we find XX and YY interactions, which induce an unwanted evolution when the switch is turned off. To obtain high-fidelity single qubit gates, these interactions should be small. The expression above shows that these can be controlled by the size of the applied field or by the size of the Co-ring exchange. With a static field of 5 T the evolution induced by H_{eff} occurs on a timescale of 0.5 μs, which is much longer than the time required for elementary gates. In the left panel of Supplementary Figure 29 we report the fidelity of an idle evolution for a pair of qubits initialized in $\frac{|01\rangle+|10\rangle}{\sqrt{2}}$ (which is one of the most error-prone states). In the absence of the residual coupling discussed above, no evolution occurs. It is worth noting that a reduction by a factor of only 2 of the Co-ring exchange is sufficient to increase this timescale by an order of magnitude, without significantly affecting the gate fidelities (red curve in Fig. S29).

Effective qubit-qubit interaction with the redox active switch

In compounds **7**, **8** and **9** an anisotropic and/or anti-symmetric qubit-switch exchange interaction leads to a form of the effective qubit-qubit coupling different from that reported in the main text (Eq. 3), which however does not hinder the feasibility of the proposed scheme. Since the qubits are symmetrically equivalent, there exist a reference frame in which their effective interaction (in a field of a few Teslas) can be described by a diagonal exchange tensor, of the form

$$H = \Gamma_x S_{1x} S_{2x} + \Gamma_y S_{1y} S_{2y} + \Gamma_z S_{1z} S_{2z},$$

apart from a constant and one-body terms. The form of the effective couplings can be rather complicated, depending on the specific qubit-switch interaction. However, it is not relevant for the feasibility of the scheme. What matters is that the unitary evolution operator (a part from an overall phase) associated to such Hamiltonian is given by

$$U(t) = \begin{pmatrix} e^{i\frac{\Gamma_z t}{2}} \cos\frac{\Gamma_x - \Gamma_y}{4} t & 0 & 0 & ie^{i\frac{\Gamma_z t}{2}} \sin\frac{\Gamma_x - \Gamma_y}{4} t \\ 0 & \cos\frac{\Gamma_x + \Gamma_y}{4} t & i\sin\frac{\Gamma_x + \Gamma_y}{4} t & 0 \\ 0 & i\sin\frac{\Gamma_x + \Gamma_y}{4} t & \cos\frac{\Gamma_x + \Gamma_y}{4} t & 0 \\ ie^{i\frac{\Gamma_z t}{2}} \sin\frac{\Gamma_x - \Gamma_y}{4} t & 0 & 0 & e^{i\frac{\Gamma_z t}{2}} \cos\frac{\Gamma_x - \Gamma_y}{4} t \end{pmatrix}$$

This still leads to a perfectly entangling gate by choosing $t = \frac{\pi}{\Gamma_x + \Gamma_y}$. The $\sqrt{\text{iSWAP}}$ gate is recovered for $\Gamma_x = \Gamma_y$, $\Gamma_z = 0$ and the $\sqrt{\text{SWAP}}$ gate for $\Gamma_x = \Gamma_y = \Gamma_z$. Therefore, the viability of the scheme does not crucially depend on the specific form of the qubit-qubit interaction. It only requires a symmetric coupling between the central Co switch and the two rings, as well as a parallel arrangement of the qubits (hence the same Zeeman energy). This is the case in the here reported complexes.

Scalability

We focus here on the effect of pure dephasing on a set of non-interacting qubits, subject to a Lindblad (Markovian) dynamics. For each qubit, initialized in the pure state $|\psi(0)\rangle = \alpha|0\rangle + \beta|1\rangle$ (corresponding to $\rho(0) = |\psi(0)\rangle\langle\psi(0)|$), the time dependence of the fidelity is given by

$$F_1 = \sqrt{|\alpha|^4 + |\beta|^4 + 2|\alpha|^2|\beta|^2 e^{-t/2T_2}} = \sqrt{1 - 2|\alpha|^2|\beta|^2\left(1 - e^{-t/2T_2}\right)},$$

where we have exploited $|\alpha|^2 + |\beta|^2 = 1$ and assumed T_M as the single-qubit dephasing time. It can be easily verified that the state maximizing the error $\varepsilon = 1 - F^2$ on a single qubit is that characterized by $|\alpha|^2 = |\beta|^2 = 1/2$. It was also recently shown[13] that the choice of $|\psi(0)\rangle^{\otimes N}$ as the initial state for N qubits is the one maximizing the N-qubits error ε_N. Its fidelity is simply the product of single qubit fidelities or, equivalently (to lowest order in ε), $\varepsilon_N = N\varepsilon_1 = \frac{N}{2}\left(1 - e^{-t/2T_2}\right)$. Finally, in the limit $t << 2T_2$ we find $\varepsilon_N \approx N\frac{t}{4T_2}$, as reported in the main text.

Below we discuss the effect of the residual qubit-qubit coupling on scalability. In the right panel of Supplementary Figure 22 we report the timescale of the unwanted evolution (T_{UE}, defined as the time required to reduce the fidelity to 0.9) as a function of the number of qubits (arranged in a linear chain).

The parameters measured by EPR in the proposed compound (blues circles) allow us only to manipulate a few qubits on a chain. Conversely, if the exchange interaction is reduced by a factor 2 (red points), many gates can be implemented even in a linear array of 8 qubits. A similar result can also be obtained by using the present compound and increasing the magnetic field from 5 to 9 T (black circles).

From an experimental point of view, we would like to stress that the proposed schemes for quantum information processing can be easily expanded to finite chains of Cr_7Ni rings, connected through the same or different central nodes (Supplementary Figure 30). In fact, this reflect one of the greatest advantage of using molecular qubits, where bottom-up supramolecular chemistry methodologies could be applied in order to obtain tailored-made assemblies in a rational way. In particular, we can envisage connecting all of the proposed 2-qubits units to form finite chains of qubits by taking advantage of molecular-programmed self-assembly methodologies. In this sense, the judicious choice and sequential combination of homo- and/or hetero-bifunctionalised {Cr_7Ni} rings together with the appropriate central node would allow us to obtain, with great control, the desired array of qubits (Supplementary Figure 31). As a proof-of-principle, in Supplementary Figure 30 we have represented several examples of assemblies that could be obtained and show the potential applicability of our design strategy to obtain assemblies of increasing complexity by combining simpler metallosupramolecular tectons.

Characterization of the ground state of $[Ru^{III}_2Co^{II}O(^tBuCO_2)_6(py)_3]$

The trigonal mixed-metal pivalate cluster $[Ru^{III}_2Co^{II}O(^tBuCO_2)_6(py)_3]$ **6** has been structurally characterised (Supplementary Figure 2a) and it total spin ground state ascertained by SQUID magnetometry (Supplementary Figure 6) and multi-frequency EPR spectroscopy (Supplementary Figure 11) as $S_{eff} = {}^1/_2$.

Magnetism: Variable-temperature (2–300 K) magnetic susceptibility measurements for **6** reveal a typical behavior of an of an octahedral, high-spin d^7 Co^{II} site ($S_{Co} = {}^3/_2$) with an important spin-orbit coupling (SOC) ($^4T_{1g}$ term in ideal O_h symmetry);[14] which is in agreement with the acetate analogue of this complex previously reported.[15] The unquenched orbital angular momentum elevates the room temperature $\chi_M T$ value to 3.11 cm^3 K mol^{-1}, well above the spin-only value for a spin-quartet. The first-order orbital angular momentum of the $^4T_{1g}$ ground state in octahedral Co(II) is coupled to the electron spin ($S = {}^3/_2$) by spin orbit coupling (SOC), therefore S and L are no longer good quantum numbers, so we must consider J, the total angular momentum. The fit in Supplementary Figure 6 was achieved via the Hamiltonian in Eq. 4 with g = 2.155, an orbital reduction parameter (σ) of -0.9, and a spin-orbit coupling constant (λ) of -171.5 cm^{-1}. The orbital reduction parameter σ = Aκ, where A = $-{}^3/_2$ and κ is the usual orbital reduction factor (0≤κ≤1), and can therefore take the values -1.5-0. The A parameter acts to simplify the calculation by creating a fictitious L =1 due to the T-P isomorphism, where the factor ($-{}^3/_2$) defines the conversion of the matrix of the angular momentum operator $\hat{l}$ within the $^4T_1$ (^4T) state to the matrix of $\hat{l}$ in the atomic p-basis.

$$\hat{H} = \beta\left(\sigma\hat{L}_{Co} + g\hat{S}_{Co}\right)H + \lambda(\sigma\hat{L}_{Co}\cdot\hat{S}_{Co}) \qquad (4)$$

$$\phi(J, m_J) = \sum_{ml\,ms} C^{J\,mJ}_{1\,ml\frac{3}{2}ms}\,|l = 1, m_l, s = \tfrac{3}{2}, m_s\rangle \qquad (5)$$

$$\phi\left(\tfrac{1}{2}, \pm\tfrac{1}{2}\right) = \left(\tfrac{1}{\sqrt{6}}\right)|1, \mp 1, \tfrac{3}{2}, \pm\tfrac{1}{2}\rangle - \left(\tfrac{1}{\sqrt{3}}\right)|1, 0, \tfrac{3}{2}, \mp\tfrac{1}{2}\rangle + \left(\tfrac{1}{\sqrt{2}}\right)|1, \pm 1, \tfrac{3}{2}, \mp\tfrac{3}{2}\rangle \qquad (6)$$

Eq. 4 gives a $J = {}^1/_2$ ground state, and can be explained by normal octahedral Co(II) behaviour made of components $|m_l, m_s>$ for $|J = {}^1/_2, m_J = \pm{}^1/_2>$ given by Clebsch-Gordon in Eq. 6, due to the antiferromagnetically coupling of the two Ru(III) ions to give S = 0. The Clebsch-Gordon coefficients can be calculated using Eq. 5.

EPR spectroscopy: Frozen solution EPR spectra of **6** at X- and Q-band at 10 K are typical of high-spin Co(II) centers with unquenched orbital angular momentum (Supplementary Figure 11). These spectra were simulated as a $S_{eff} = {}^1/_2$ giving rhombic g = (5.61, 4.05, 2.77); which is similar to the parameters of the acetate analogue of this complex previously reported.[16] The average g-value of 4.14 is comparable to the calculated value of 4.19 for the $J = {}^1/_2$ ground state in the fitting of the magnetic data. Coupling of the electron spin to the $I = {}^7/_2$ nuclear spin of ^{59}Co (100% natural abundance) is evident on g_1 and g_2 at X-band. Simulation of this spectrum gave A = (180, 138, 20) × 10^{-4} cm^{-1}.

Supplementary References

1. Larsen, F. K. *et al.* Synthesis and Characterization of Heterometallic {Cr_7M} Wheels. *Angew. Chem., Int. Ed.*, **42**, 101–105 (2003).
2. Timco, G. A. *et al.* Engineering the coupling between molecular spin qubits by coordination chemistry. *Nat. Nanotechnol.* **4**, 173–178 (2008).
3. Constable, E. C. *et al.* Expanded ligands: bis(2,2':6',2''-terpyridine carboxylic acid)ruthenium(II) complexes as metallosupramolecular analogues of dicarboxylic acids. *Dalton Trans.* **38**, 4323–4332 (2007).
4. Nowell H., Barnett S. A., Christensen K. E., Teat S. J., Allan D. R. I19, the small-molecule single-crystal diffraction beamline at Diamond Light Source. *J. Synchrotron Radiat.* **19**, 435–441 (2012).
5. Sheldrick, G. M. SADABS Empirical absorption correction program based upon the method of Blessing (University of Göttingen, 1995).
6. Blessing, R.H. An empirical correction for absorption anisotropy. *Acta Crystallogr.* **A51**, 33–38 (1995).
7. Sheldrick, G. M. A short history of SHELX. *Acta Crystallogr.* **A64**, 112–122 (2008).
8. Sheldrick, G. M. A short history of SHELX. *Acta Crystallogr.* **A71**, 3–8 (2015).
9. PLATON, A Multipurpose Crystallographic Tool (Utrecht University, Utrecht, The Netherlands, 2008).
10. Connelly, N. G. and Geiger W. E. Chemical Redox Agents for Organometallic Chemistry. *Chem. Rev.* **96**, 877–910 (1996).

11. Stoll, S. and Schweiger, A. EasySpin, a comprehensive software package for spectral simulation and analysis in EPR. *J. Magn. Reson.* **178**, 42–55 (2006).

12. Jeschke, G. *et al.* DeerAnalysis2006–a comprehensive software package for analyzing pulsed ELDOR data. *Appl. Magn. Reson.* **30**, 473 (2006).

13. Jing, J. and Hu, X. Scaling of decoherence for a system of uncoupled spin qubits. *Sci. Rep.* **5**, 17013 (2015).

14. Lloret, F., Julve, M., Cano, J., Ruiz-García, R. and Pardo, E. Magnetic properties of six-coordinated high-spin cobalt(II) complexes: Theoretical background and its application. *Inorg. Chim. Acta* **361**, 3432–3445 (2008).

15. Ohto, A., Sasaki, Y. and Ito, T. Mixed-Metal Trinuclear Complexes Containing Two Ruthenium(III) Ions and a Divalent Metal Ion, $[Ru_2M(\mu_3\text{-}O)(\mu\text{-}CH_3COO)_6(L)_3]$ (M = Mg, Mn, Co, Ni, Zn; L = H_2O, Pyridine). *Inorg. Chem.* **33**, 1245–1246 (1994).

16. Kobayashi, H. *et al.* Magnetic Properties of Some Triangular Trinuclear Complexes of $[Ru_2M(\mu\text{-}CH_3COO)_6(\mu_3\text{-}O)(py)_3]^{n+}$ with a Paramagnetic Ion M. *Bull. Chem. Soc. Jpn.* **69**, 3163–3172 (1996).
